# Digital Twin-Based Technical Research on Comprehensive Gear Fault Diagnosis and Structural Performance Evaluation

**DOI:** 10.3390/s25092775

**Published:** 2025-04-27

**Authors:** Qiang Zhang, Zhe Wu, Boshuo An, Ruitian Sun, Yanping Cui

**Affiliations:** 1 Key Laboratory of Vehicle Transmission, China North Vehicle Research Institute, Beijing 100072, China; 12116325@bjtu.edu.cn; 2 School of Mechanical Engineering, Hebei University of Science and Technology, Shijiazhuang 050018, China; 15511381917@163.com (B.A.); iknow_66@163.com (R.S.); cuiyp@hebust.edu.cn (Y.C.)

**Keywords:** dynamic data, artificial intelligence, fault diagnosis, gearbox, integrated form-performance

## Abstract

In the operation process of modern industrial equipment, as the core transmission component, the operation state of the gearbox directly affects the overall performance and service life of the equipment. However, the current gear operation is still faced with problems such as poor monitoring, a single detection index, and low data utilization, which lead to incomplete evaluation results. In view of these challenges, this paper proposes a shape and property integrated gearbox monitoring system based on digital twin technology and artificial intelligence, which aims to realize real-time fault diagnosis, performance prediction, and the dynamic visualization of gear through virtual real mapping and data interaction, and lays the foundation for the follow-up predictive maintenance application. Taking the QPZZ-ii gearbox test bed as the physical entity, the research establishes a five-layer architecture: functional service layer, software support layer, model integration layer, data-driven layer, and digital twin layer, forming a closed-loop feedback mechanism. In terms of technical implementation, combined with HyperMesh 2023 refinement mesh generation, ABAQUS 2023 simulates the stress distribution of gear under thermal fluid solid coupling conditions, the Gaussian process regression (GPR) stress prediction model, and a fault diagnosis algorithm based on wavelet transform and the depth residual shrinkage network (DRSN), and analyzes the vibration signal and stress distribution of gear under normal, broken tooth, wear and pitting fault types. The experimental verification shows that the fault diagnosis accuracy of the system is more than 99%, the average value of the determination coefficient (R^2^) of the stress prediction model is 0.9339 (driving wheel) and 0.9497 (driven wheel), and supports the real-time display of three-dimensional cloud images. The advantage of the research lies in the interaction and visualization of fusion of multi-source data, but it is limited to the accuracy of finite element simulation and the difficulty of obtaining actual stress data. This achievement provides a new method for intelligent monitoring of industrial equipment and effectively promotes the application of digital twin technology in the field of predictive maintenance.

## 1. Introduction

With the advent of new-generation information and communication technology and artificial intelligence, traditional manufacturing is advancing towards IT-based intelligent manufacturing [1]. Gears, known for their precise transmission ratio, high transmission efficiency, and robust load-bearing capacity, are widely used in mechanical manufacturing, aerospace, and other fields. However, during gear operation, due to complex working conditions and the difficulty of obtaining real-time performance data, various failures are prone to occur. The performance of high-end equipment often largely depends on the performance of key components (stress–strain, temperature, fatigue), with increasing demands on these performances. Traditional maintenance methods, typically post-event maintenance and periodic maintenance, exhibit certain lagging characteristics. Moreover, traditional monitoring systems, such as rotating machinery fault diagnosis and prediction systems, typically convert parameters into a large number of vibration signals for indication. The sheer volume of signals makes it challenging for monitoring systems to accurately and promptly diagnose system faults. Current traditional gearbox monitoring methods have the following main shortcomings: (1) Insufficient monitoring intensity, making it difficult to capture the gear condition accurately in real-time; (2) Limited detection metrics, lacking comprehensive evaluation of multidimensional performance; (3) Low data utilization, failing to fully leverage the potential of the collected data.

To address these issues, this paper proposes a new solution: integrating finite element analysis, artificial intelligence, fault detection, and visualization technology to construct a unified shape and performance digital twin model for rotating machinery. By utilizing a small number of sensors, real-time analysis and characterization of gear faults (such as pitting, wear) and structural performance (such as stress–strain, temperature, fatigue) can be achieved. This method enables monitoring of gear operating conditions and visualizes real-time fault status and performance data of the equipment. The integrated shape and performance digital twin monitoring method holds significant practical value for utilizing information technology in the monitoring and prediction of rotating machinery.

Current fault diagnosis technology plays a crucial role in industrial and scientific fields. Advances in intelligent systems and machine learning are driving the innovation and enhancement of fault diagnosis techniques [2]. Diagnostic methods have evolved from rule-based expert systems to analytical model-based approaches, and now further to data-driven deep learning models, making fault diagnosis more intelligent and accurate.

Data-driven fault diagnosis methods diagnose faults by analyzing actual data acquired from the system. The fundamental idea of data-driven fault diagnosis is to utilize machine learning, data mining, and statistical analysis techniques to discover patterns and regularities from sensor data, enabling the detection and diagnosis of system faults. Khorram et al. [3] proposed an end-to-end approach using raw time-domain features collected from accelerometers as input. This approach detects bearing faults through a novel time series prediction algorithm, employing equivalent time series as input to a Convolutional Long Short-Term Memory Recurrent Neural Network (CRNN), achieving the highest accuracy in bearing fault detection in the shortest time. A. Krizhevsky et al. [4], in their study on image classification using Convolutional Neural Networks (CNN) for the ImageNet Large Scale Visual Recognition Challenge (ILSVRC), increased the accuracy from 74.2% to 83.6% compared to traditional intelligent methods. Large deep convolutional neural networks can achieve breakthrough results on challenging datasets, and the depth of the network is crucial for these achievements. Lei Yaguo et al. [5] proposed an innovative health monitoring strategy using Deep Neural Networks (DNN) to automatically extract fault features from the frequency domain signals of mechanical equipment. This method is suitable for handling complex monitoring and diagnosis tasks, enabling more precise assessment of mechanical equipment health. Data-driven fault diagnosis methods do not rely on specific expert knowledge or a deep understanding of the internal workings of the system. They offer better real-time and automation capabilities for complex situations and can adapt to various types of systems and fields.

Grieves first proposed the concept of digital twin in 2013, exploring its definition and how digital twin technology can mitigate unpredictable and adverse emergent behaviors in complex systems [6]. The digital twin concept integrates optimal computational models to continuously predict the health status, remaining useful life, and mission success probability of vehicles or systems [7]. H. Uhlemann et al. [8] combined digital twin technology with mechanical manufacturing processes to construct a digital twin system with real-time monitoring and evaluation functions. Seshadri et al. [9] integrated digital twin technology with fault diagnosis algorithms, using genetic algorithm optimization programs to effectively predict the location, size, and direction of damage, providing an effective method for health monitoring of aircraft structures. Digital twin technology has been applied in various fields, including data-driven, human–machine interaction, and mechanical manufacturing domains. Physical methods primarily involve numerical simulations, with the finite element method being one of the most practical numerical simulation techniques. It can serve as a data-driven method for digital twin systems, and its feasibility has been validated [10]. T. Moi et al. [11] introduced a new method for state monitoring of a small articulated boom crane based on digital twin technology. The crane’s digital twin is simulated in real-time within a nonlinear finite element program, providing a new approach to monitor and predict equipment status through digital twin technology. Feng et al. [12] used the digital twin five-dimensional model to realize the operation monitoring function of CNC machine tools, which combined two-dimensional data chart and three-dimensional motion simulation. Zhang et al. [13] established a digital twin monitoring system for mine hoist’s crown wheel through the construction of virtual space, data transmission and application modules, and verified through experiments that the designed prediction model could meet the requirements for monitoring the structure performance of the crown wheel. Although significant progress has been made in current research, real-time display of high-fidelity simulation models has not yet been achieved. Given current computational efficiency, this capability is limited to the early stages of system design, unable to fully unleash the potential of digital twin systems [14].

Based on the aforementioned research findings and challenges, researchers have discovered that data-driven models not only enable the reuse of training resources but also have lower computational costs compared to high-fidelity simulations. Additionally, with the continuous development of artificial intelligence technology in the field of digital twins, the realization of digital twin systems based on high-fidelity simulation models has become possible. Compared with the previous research mainly relying on the finite element method, which is applied to the condition monitoring and modeling of traditional mechanical systems, these methods are mostly limited by the high amount of calculation and are difficult to realize the real-time update of high fidelity models. Recently, scholars have broken the bottleneck that the traditional high fidelity models are limited by the computational efficiency and difficult to run in real time. Through the introduction of agent modeling and intelligent algorithms, especially breakthroughs in new fields such as human–computer interaction and biomechanical risk analysis, they have laid a solid foundation for the application of digital twin technology in complex systems, and have made progress in exploring the application of high fidelity digital twin systems in a more complex and multi-source system environment. Conforti et al. [15] measured and identified biomechanical risks in load-handling tasks using wearable systems and machine learning methods. The results demonstrated that real-time posture analysis through wearable devices and biomechanical models, combined with machine learning algorithms, can effectively identify and prevent unsafe postures in the workplace, thereby reducing the risk of musculoskeletal disorders. Chakraborty et al. [16] explored the concept of digital twins using a discrete damped dynamic system and specifically examined the application of Gaussian process surrogate models in digital twin technology. They proposed future research directions for digital twins, including the application of surrogate model-based digital twins in big data processing, machine learning integration, multi-degree-of-freedom systems, nonlinear systems, and long-term predictions.

To sum up, although existing studies have explored the application of digital twin technology in different fields, and some work has also proposed proxy models or multi-level architectures, most studies still focus on a functional dimension or a modeling method, lacking effective integration of “high fidelity real-time visualization” integration from the system architecture level. Especially in the aspect of “implementation of digital twin system based on high-precision model”, there is still a lack of complete path to clearly explain its implementation mechanism. Therefore, in order to solve the above problems, starting from the service scenario, a more complete digital twin monitoring framework at the system level is constructed, and the whole process of digital twin in fault diagnosis and performance prediction is realized. In terms of modeling method, the system integrates the finite element multi physical field simulation and Gaussian process regression (GPR) surrogate model, takes into account the high accuracy and efficiency of stress prediction, and introduces wavelet transform and depth residual shrinkage network (DRSN), which significantly improves the fault identification performance under complex working conditions. In terms of visualization, the three-dimensional real-time cloud image rendering of gear fault state is realized based on unity3d platform, which enhances the interactivity and visual expression of the system, and breaks through the limitation that most existing researches only stay in two-dimensional or static display. The system can effectively deal with the problems existing in the current gear monitoring, such as insufficient monitoring coverage, single detection index and low data utilization, which lead to incomplete evaluation results. This paper presents a new solution, which is the digital twin gear monitoring system with the integration of shape and property. In this study, the system can realize real-time gear condition monitoring and performance evaluation by building a digital twin framework based on high fidelity simulation model, combining data-driven model and artificial intelligence algorithm. The research goal of this paper is to develop a gear digital twin monitoring system with high real-time and accuracy, in order to improve the accuracy and response speed of gear fault diagnosis. The expected contributions include: (1) a digital twin gear monitoring system framework with shape and property integration is proposed; (2) The application and verification of digital twin technology in gear fault diagnosis are realized; (3) Provide a set of scalable solutions to promote the further development of digital twin technology in the field of mechanical equipment monitoring.

## 2. Integrated Form-Performance Gearbox Monitoring System Framework

### 2.1. Integrated Form-Performance Digital Twin Framework

The concept of the digital twin was first introduced by David Gelernter in 1991 and later popularized in manufacturing by Dr. Michael Grieves in 2002. Subsequently, the U.S. Air Force Research Laboratory, seeking to reduce aircraft maintenance costs, required a predictive maintenance method to minimize downtime and maintenance costs, and aimed to improve aircraft service life prediction methods. This need from the Air Force significantly propelled the development of the digital twin concept [17].

**Figure 1:**
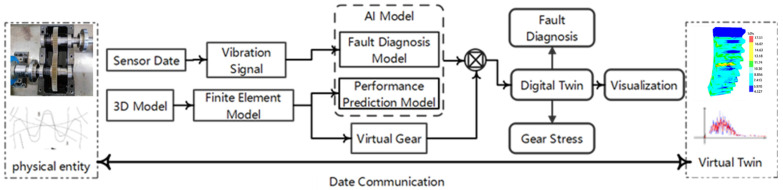
Structure of the Digital Twin Model.

As the demand for digital twin applications continues to grow, the data exchange between the physical entity and the virtual twin has become a focal point of research. Figure 1 illustrates the structure of the digital twin model, which consists of the physical entity, the virtual twin, twin data, services, and the connections between them.

Physical Entity: As the foundation, it is primarily used for state analysis to ensure proper operation of the equipment. The data from the physical entity includes various operational states and performance metrics, collected through sensors.

Virtual Twin: This is a virtual representation of the physical entity, mapping it from four perspectives: geometry, physics, behavior, and rules. These four perspectives—geometric model, physical model, behavioral model, and rules model—together constitute the complete representation of the virtual twin.

Twin Data: This is the core of the digital twin model, containing the data and dynamic information that drive the entire system’s operation. Twin data and information are kept up-to-date in real time, reflecting the system’s current state and future trends.

Services: This comprises various functionalities available to users, including equipment monitoring, fault warning, performance optimization, etc. Services use software to present system data to operators in an intuitive manner to meet their needs.

Connections: These are the interactive pathways between the physical entity, virtual twin, and services, ensuring the exchange and collaboration of information between the twin data, physical entity, and virtual twin. Connections can be network-based or data interfaces, and appropriate connection methods are employed to ensure the system’s efficient operation.

### 2.2. Integrated Shape and Performance Gearbox Monitoring System Architecture

**Figure 2:**
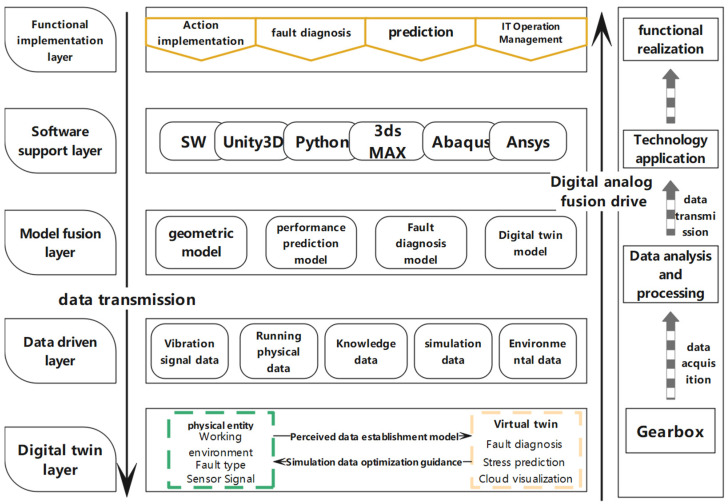
Architecture Diagram of the Integrated Shape and Performance Gearbox Monitoring System.

The goal of the integrated shape and performance gearbox monitoring system based on digital twin technology is to achieve intelligent fault diagnosis, performance prediction, and visual monitoring of the gears within the gearbox. Figure 2 illustrates the architecture of the integrated shape and performance gearbox monitoring system. Based on digital twin technology, this architecture comprises several layers: the Functional Implementation Layer, Software Support Layer, Model Fusion Layer, Data-Driven Layer, and Digital Twin Layer.

(1) Functional Service Layer

The Functional Service Layer aims to provide users with a high-quality interactive experience through a simple and intuitive service system. It includes functionalities such as action implementation, fault diagnosis, performance prediction, and maintenance management.

(2) Software Support Layer

The Software Support Layer comprises the software required for the digital twin monitoring system. The development engine used is Unity3D. SolidWorks 2020 is employed to build 3D models of the gearbox and gears. The Xdreamer-V22 plugin is utilized to perform basic gear meshing actions, and 3Ds Max is used for texturing and rendering the models. Abaqus 2023 is used for thermo-fluid-solid coupling simulation and analysis of the gears. Python 2023.2.1 and C# are used to implement the prediction models and scripts for the monitoring system.

(3) Model Fusion Layer

The Model Fusion Layer involves creating digital mapping models of the gearbox to drive physical data in virtual space. The integrated shape and performance gearbox control system achieves its functionality by merging da-ta-driven approaches with digital mapping models, systematically linking various models together through specific relationships.

(4) Data-Driven Layer

The Data-Driven Layer provides the input for the Model Fusion Layer and is the core of the data-driven system. Its function is to manage and store data related to various parameters of the gears (such as module, size, material), operational status data (such as stress, rotational speed, load), simulation data (such as stress, temperature), and environmental data (such as ambient temperature, oil proportion). It also includes, but is not limited to, vibration data for gear fault diagnosis, finite element simulation data, and maintenance knowledge data.

(5) Digital Twin Layer

The Digital Twin Layer includes both the physical entity of the gear and its virtual twin. This layer forms the foundation of the integrated shape and performance gearbox model and is used to interpret the composition and assembly behavior rules of the gearbox structure.

## 3. Construction Method for the Integrated Shape and Performance Gearbox Control System

### 3.1. Virtual-Physical Mapping

Gears operate under variable and uncertain conditions while enduring long-term loads, which inevitably leads to degradation or faults. Based on the five-dimensional digital twin model described, an integrated shape and performance gearbox monitoring system is constructed. This system achieves real-time monitoring of gear faults by collecting real-time operational data from the gearbox, ensuring the safety of the equipment during service, and promptly identifying and resolving issues to minimize unnecessary economic losses.

(1) Physical Space

**Figure 3:**
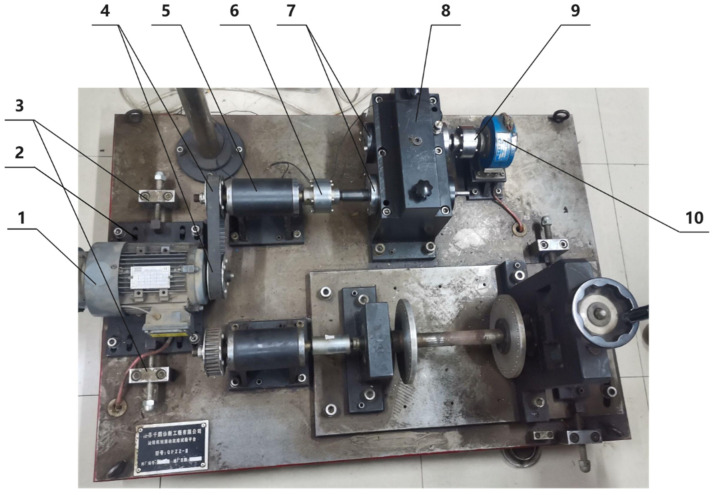
Physical Entity. 1. Three-Phase AC Frequency Converter Motor; 2. Motor Mounting Base; 3. Motor Position Adjustment Bolt; 4. Synchronous Pulley; 5. Drive Shaft Bearing; 6. Pin Coupling; 7. Drive Gear Shaft; 8. Gearbox; 9. Motor Shaft Coupling; 10. Magnetic Powder Torque Meter.

Figure 3 shows the physical entity of the QPZZ-II test rig. The physical space primarily consists of the physical entity, which is composed of various components within the QPZZ-II test rig. This test rig can simulate and analyze various states and vibration conditions of gears. Different faults are simulated by assembling various loads, installing different faulty gears, and adjusting the positions of components.

**Figure 4:**
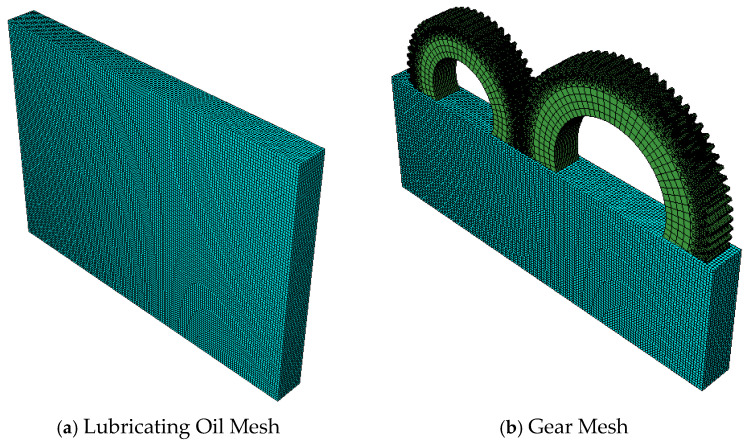
Finite Element Model Mesh.

Due to the high requirements for mesh division in the finite element simulation model of gears, the meshing functionality of Abaqus cannot meet the high-precision demands of the simulation. To improve simulation accuracy, a more specialized finite element preprocessing software, HyperMesh, was used to complete the meshing of the finite element model. After meshing in HyperMesh, the model was saved as an .inp file and imported into Abaqus. Table 1 shows the number of meshes for each component, and Figure 4 displays the finite element model mesh.

**Figure 5:**
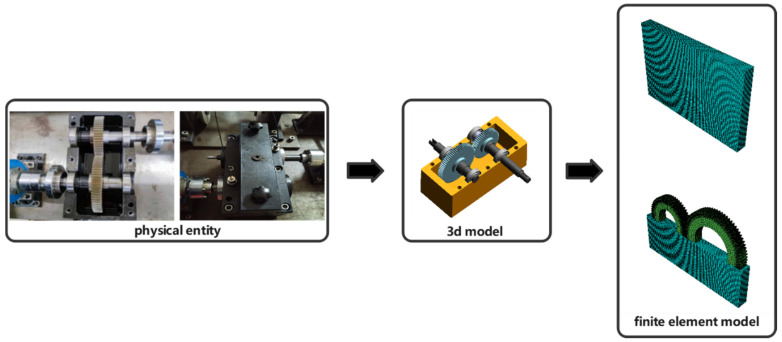
Internal Structure and 3D Model of the Gearbox.

Table 2 lists the parameters of key components. Figure 5 shows the internal structure and 3D model of the gearbox. The QPZZ-II test rig involves several components related to the gearbox, including a three-phase AC frequency converter motor, a pin coupling, the gearbox, a full-steel anti-vibration base, and a magnetic powder torque meter. The gearbox consists of two straight-toothed cylindrical gears, an upper and lower housing, an input shaft, an output shaft, and end bearings. The operation of the gearbox system is powered by the three-phase AC frequency converter motor. The power is transmitted through a belt and coupling to the small gear inside the gearbox, which then transfers power to the large gear to drive the load. For the research on the gearbox, a geometric model consistent with the physical object is established based on the manufacturer’s drawings and standards. This ensures the accuracy of the virtual twin and simulation data.

(2) Virtual Twin Space

The virtual twin space serves two main functions: first, it provides a three-dimensional mirror of the physical entity of the gearbox; second, it performs digital analysis of the state and performance predictions during the operation of the physical entity. This can be explained through the construction of the virtual twin, simulation models (including load combination models, dynamic simulation models, and operational simulation models), fault diagnosis models, and surrogate models.

First, the construction of the virtual twin involves determining the 3D modeling plan based on the geometric dimensions and shapes of the gearbox, gears, and bearings from the QPZZ-II test rig. Using UG 12.0 software, a 1:1 scale 3D model is created and exported in STL triangular mesh format. To reduce the load on subsequent CPU and GPU rendering, a simple optimization is performed in 3Ds Max software. The model is then exported in FBX format, and with coordinate transformation, it can be restored in Unity 3D 2022.

**Figure 6:**
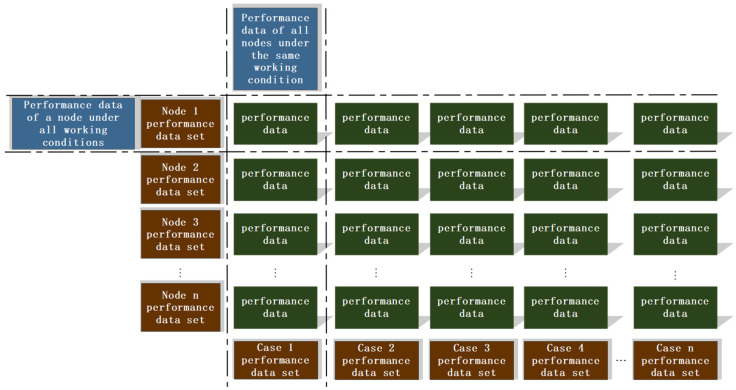
Surrogate Model Construction Flowchart.

Figure 6 illustrates the process flow for constructing the surrogate model. Using Abaqus software, thermal-flow-solid coupling simulation models for gears under different loads are created, and finite element analysis is performed for gear rotation cycles under various loads. This analysis provides dynamic stress data for each node within the gear rotation cycle, which serves as the training dataset for the surrogate model. The purpose of constructing the surrogate model is to enhance the computational efficiency of the prediction model while ensuring accuracy. Surrogate models based on simulation data are built using KNN (k-Nearest Neighbors) and GPR (Gaussian Process Regression) algorithms to predict based on input analysis. Once gear faults are identified, sensor-acquired operational information is transmitted to the condition-extension surrogate model to invoke the stress surrogate model set for the current condition. Subsequently, by manually inputting the angle into the integrated shape and performance gear-box monitoring platform, the surrogate model calculates and outputs the stress data at various points of the gear for the given angle.

Firstly, comprehensively sort out the performance data of each node under different working conditions, horizontally sort out the data set of node dimension (such as the performance data set of node 1 to node n under all working conditions), vertically integrate and build the data set of condition dimension (such as the performance data set of all nodes from condition 1 to condition n respectively), provide data support for subsequent agent model building, and then use these structured data to complete the training and construction of agent model, so as to realize the effective simulation and prediction of complex system performance.

The pseudo code of the proxy model is shown in Algorithm 1. The system receives the angle input from unity through TCP socket, identifies the angle parameters, and uses the pre trained proxy model array based on KNN and GPR to predict the stress value of each node of the gear. The generated stress sequence is encoded in JSON format and sent back to unity engine for visualization and monitoring.
**Algorithm 1.** Proxy Model Partial Pseudo CodeInput: Angle command θ_input from Unity (format: ‘feaXXX’) Output: Predicted stress sequence y_pred (JSON encoded) Load surrogate model array Func [1:26510] using joblib Initialize TCP socket server and bind to port 8000 Start listening and accept client connection from Unity While system is running:  Receive message data_unity from Unity  If ‘fea’ in data_unity:    Strip ‘fea’ and parse θ_input ← float(data_unity)    Initialize empty list y_pred ← []    For j = 1 to 26,510 do:      y_j ← Func[j].predict([[θ_input]])      Append round(y_j, 5) to y_pred      Encode y_pred to JSON format      Send y_pred to Unity through socket

For the construction of the fault diagnosis model, vibration data from different faulty gears are obtained by simulating operations on the QPZZ-II test rig. This data is used as training data for the fault diagnosis model. The model is trained using wavelet transform-based algorithms and deep residual shrinkage network methods. Vibration signals collected from various sensors arranged on the physical gearbox are input in real-time into the fault diagnosis model to detect gear faults.

(3) Virtual-Physical Mapping Process

The pseudo code of virtual real mapping is shown in Algorithm 2. The system initializes the proxy model server and GUI, and then dynamically loads the corresponding GPR model set according to the identified fault type. After receiving unity’s angle command, the system uses the pre trained agent model to calculate the node stress prediction, determine the maximum stress, and transmit the results to unity for real-time 3D visualization. At the same time, the GUI updates the corresponding stress evolution curve to realize the real-time information physical interconnection of the digital twin platform.

**Figure 7:**
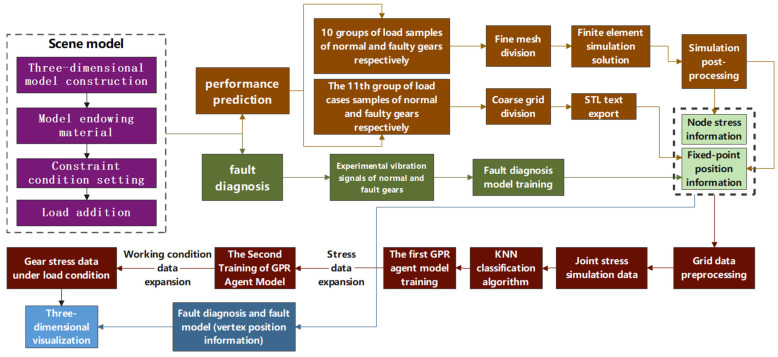
Virtual-Physical Mapping Process Flowchart.

**Figure 8:**
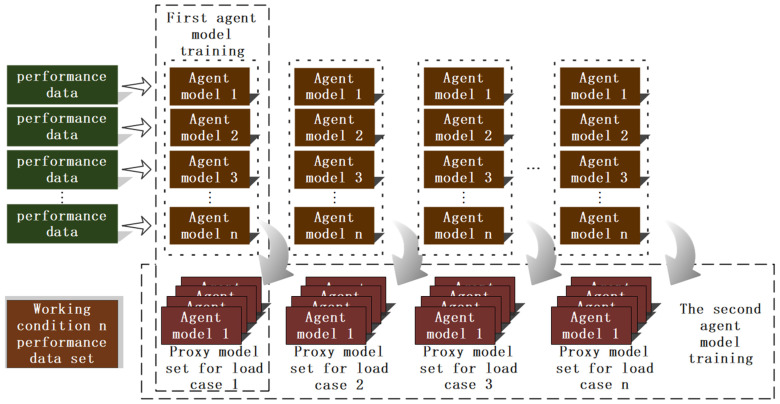
Proxy Model Flowchart with Load Condition Prediction Values and Included Proxy Model Set as Output.

Figure 7 is Virtual-Physical Mapping Process Flowchart. Unity 3D is used as the development engine to achieve the mapping of the virtual twin to the physical entity through sensor signals. The construction of fault types and stress cloud map visualization modules involves obtaining vibration data from various faults using the QPZZ-II test rig, which is used as the fault diagnosis training dataset. Algorithms are then employed to train the fault diagnosis model based on this data. For stress prediction, Abaqus is utilized to obtain stress data under different load conditions and angles, forming the stress prediction training dataset. The KNN algorithm is first used to classify stress data for coarse grid nodes based on neighboring sample data from fine grid simulations. The GPR algorithm is then applied to perform two stages of surrogate model training based on the classified dataset. The training results are transmitted to Unity 3D via Socket for three-dimensional visualization of the stress cloud map on the virtual twin. The detailed virtual-physical mapping process is as follows:

(1) Condition Determination: Based on historical operational data of the gearbox, determine the load magnitude within the gear rotation cycle. Obtain 10 sets of different load conditions within the historical load range. Simulate these 10 conditions in Abaqus and collect simulation data, with each condition providing 500 frames of stress data covering one full 365° rotation of the gear. In addition to the 10 different condition simulation models, an 11th condition model is added.
(2) Mesh Generation and Refinement: After preprocessing the first 10 conditions by assigning material properties and setting boundary conditions, refine the mesh to provide more accurate simulation results. For the 11th condition model, perform a coarse mesh generation. Export the model in .STL format and use a Python script to remove duplicate vertex information from the triangular facets of the model. Export the unique vertex coordinates and vertex indices as two text files to achieve mesh dimensionality reduction.
(3) Construction of Fault Diagnosis and Performance Prediction Algorithms: For fault diagnosis, use experimental data to train the fault diagnosis model. For performance prediction, first apply the KNN algorithm to average the stress of neighboring mesh nodes, and then use the GPR algorithm to construct a GPR surrogate model. Figure 8 illustrates the surrogate model workflow with load condition predictions and the associated surrogate model sets as outputs. The GPR surrogate model will train two prediction models: the first model predicts node stress based on 500 frames of stress data with angle as input, and the second model predicts load conditions and includes the surrogate model sets based on the sensor data. During the offline phase, the models are validated, and during the online phase, the fault diagnosis model and GPR surrogate model can be used for rapid diagnosis and prediction. The fault diagnosis model can be exported as a .PTH format file, while the two surrogate models for each condition and node will be packaged into .PKL format files.

(4) Loading PTH and PKL Files and Running the Main Program: Integrate the two model files into the integrated gear monitoring system. Diagnose fault types based on real-time sensor data and obtain the current load condition’s gear stress prediction set using the GPR proxy model based on fine mesh simulation data.
(5) Fault Model and Stress Cloud Map Visualization: Use a Python main program combined with Socket communication to interact with Unity3D using C# scripts. Finally, display the three-dimensional fault model of the gear and the corresponding stress cloud map visualization in Unity3D.

**Algorithm 2.** Process Pseudo Code: pseudo code of virtual real mapping partInput: Angular command θ_input from Unity, Fault type Fault_Type Output: Predicted stress sequence y_pred, Maximum stress σ_max Initialize GUI and start Socket server (e.g., port 8000/8001) Load the corresponding GPR surrogate model array Func [1:N] based on Fault_Type While system is running:  Wait for incoming angle command from Unity marked as “feaXXX”  Parse θ_input into a float θ  Initialize empty list y_pred ← []  For i = 1 to N do:    y_i ← Func[i].predict(θ)    Append round(y_i, 5) to y_pred  σ_max ← max(y_pred)  Encode y_pred as JSON and send it to Unity via Socket  Append σ_max to GUI time-series plot  Update the GUI with current stress curve and value display

### 3.2. Data Interaction

**Figure 9:**
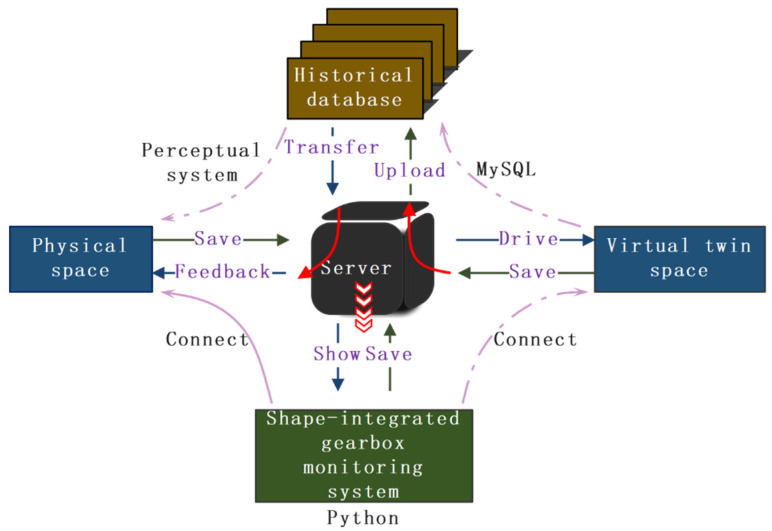
Data Interaction Flowchart of the Integrated Physical-Virtual Gearbox Monitoring System.

Figure 9 shows the data interaction flowchart for the integrated physical-virtual gearbox monitoring system. The sensing system (sensors, data collectors, decoders) transmits data to the server via serial communication. Socket communication, based on the TCP/IP protocol, imports the data collected from the physical entity into the database in real-time, storing it as historical data. This data drives the virtual space for fault diagnosis and performance prediction, and the results are displayed in real-time in the visualization module of the gearbox monitoring system. The virtual twin space uses Socket communication to store the displayed data on the server, and then uploads the data to the database. Historical data in the database can be called upon from the server, which is then presented again in the integrated physical-virtual gearbox monitoring system, and can also be fed back to the physical entity for decision-making.

## 4. Artificial Intelligence Models

### 4.1. Gaussian Process Regression

Surrogate models, also known as metamodels or proxy models, are used to approximate the mathematical relationship between inputs and outputs based on a limited set of sample information. These models are widely applied in the design and optimization of complex structures and multidisciplinary systems. In digital twin systems, surrogate models enable real-time computation of physical models. Common surrogate models used in structural optimization include Polynomial Response Surfaces (PRS), Kriging (KRG), Radial Basis Functions (RBF), Support Vector Regression (SVR), and Moving Least Squares (MLS). In this study, Gaussian Process Regression (GPR) is utilized to construct the surrogate model.

Gaussian Process Regression (GPR) is a non-parametric regression method framed within Bayesian probability, which is notably effective in handling nonlinear data [18,19]. This section explains how to develop an AI-based stress prediction model using GPR to forecast stress variations in gears in real-time.

When fitting a Gaussian Process Regression (GPR) model, the function $f\left(x\right)\sim g\left(m\left(x\right),K\left(x,x^{\prime }\right)\right)$ a being fitted can be represented as:

(1) $$\left\{\begin{array}{l} m(x)=E[(f(x))]\\ k(x,x^{\prime })=E[(f(x)-m(x))(f(x^{\prime }-m(x^{\prime })))] \end{array}\right.$$

where x is the input vector;

$m\left(x\right)$ is a given mean function;

$K\left(x,x^{\prime }\right)$ is the covariance function of the Gaussian distribution f with respect to any two points x and $x^{\prime }$.

Assume the operational relationship between the input vector x of the GPR model and the target observations y is given by:

(2) $$y_{i}=f\left(x_{i}\right)+\varepsilon$$

where $f\left(x_{i}\right)$ is an arbitrary function;

ε is regression residual (noise) satisfies $\varepsilon \sim N\left(0,\sigma^{2}\right)$.

In this study, the operational parameters are identified as input vectors, and the stress values are identified as output vectors.

In Gaussian Process Regression, the covariance between any two points x and $x^{\prime }$ can be represented using a kernel function $K\left(r\right)$. A commonly used kernel function is the RBF kernel function:

(3) $$K\left(r\right)=\sigma^{2}exp\left(-\frac{\left\|r^{2}\right\|}{2l^{2}}\right)$$

Periodic kernel function:

(4) $$K\left(r\right)=\sigma^{2}exp\left(-\frac{2}{l^{2}}\sin\left(\frac{\pi }{p}\right)r\right)$$

where r is the distance measure between x and $x^{\prime }$ in Euclidean space;

σ, l and p is hyperparameters in kernel functions.

The prior distribution of the noise observation values is considered as:

(5) $$y\sim N\left(0,K\left(x,x\right)+\sigma_{N}^{2}I_{N}^{2}\right)$$

where $K\left(x,x\right)$ represents the N×N order covariance matrix, and $I_{N}$ denotes the identity matrix.

The joint prior distribution of the observations y and the predictions $f^{*}$ is given by:

(6) $$\left[\begin{array}{c} y\\ f^{*} \end{array}\right]\sim N\left(0,\left[\begin{array}{cc} K\left(x,x\right)+\sigma_{N}^{2}I_{N}^{2} & K\left(x,x^{*}\right)\\ K\left(x^{*},x\right) & K\left(x^{*},x^{*}\right) \end{array}\right]\right)$$

To verify, the key equations of Gaussian Process Regression are derived based on Bayes’ theorem:

(7) $$p\left(f^{*}|x,y,x^{*}\right)\sim N\left[\bar{f^{*}},cov\left(\bar{f^{*}}\right)\right]$$

In the above equation:

(8) $$\bar{f^{*}}=m\left(x_{*}\right)+K\left(x^{*},x\right){\left(K+\sigma^{2}+\sigma^{2}\cdot I_{N}\right)}^{-1}\left(y-m\left(x_{*}\right)\right)$$

(9) $$cov\left[\bar{f^{*}}\right]=K\left(x^{*},x^{*}\right)-K\left(x^{*},x\right){\left(K+\sigma^{2}I_{N}\right)}^{-1}K\left(x,x^{*}\right)$$

where $\bar{f^{*}}$ represents the predictive mean of the function $f\left(x\right)$;

$cov\left[\bar{f^{*}}\right]$ represents the variance of the function $f\left(x\right)$.

For all nodes in the finite element model, a GPR model needs to be constructed for each node to predict the corresponding stress data.

(10) $$\left\{\begin{array}{c} p\left(f^{*\left(1\right)}|x^{\left(1\right)},y^{\left(1\right)},x^{*\left(1\right)}\right)\sim N\left[{\bar{f^{*}}}^{\left(1\right)},cov\left({\bar{f^{*}}}^{\left(1\right)}\right)\right]\\ p\left(f^{*\left(2\right)}|x^{\left(2\right)},y^{\left(2\right)},x^{*\left(2\right)}\right)\sim N\left[{\bar{f^{*}}}^{\left(2\right)},cov\left({\bar{f^{*}}}^{\left(2\right)}\right)\right]\\ \vdots \\ p\left(f^{*\left(m\right)}|x^{\left(m\right)},y^{\left(m\right)},x^{*\left(m\right)}\right)\sim N\left[{\bar{f^{*}}}^{\left(m\right)},cov\left({\bar{f^{*}}}^{\left(m\right)}\right)\right] \end{array}\right.\;$$

Compared to other surrogate modeling algorithms, Gaussian Process Regression (GPR) exhibits strong robustness to training data and allows for the evaluation of results based on predicted deviations. As a powerful nonlinear regression algorithm, GPR is well-suited for handling high-dimensional and large-scale data. It enhances the rapid responsiveness of computational models, addressing the challenges of lengthy computation times and high costs.

The effectiveness of the conformal integration system is verified by comparing the off-line finite element simulation stress and the real-time predicted gear stress in the process of gear rotation after the gear fault is determined. After the hardware obtains the gear vibration signal and analyzes the gear fault, then Unity3D obtains the gear fault type, and renders the gear digital twin model corresponding to the fault in real time, so as to build a real-time data set and model consistent with the fault. According to the common fault types, the gear meshing of normal gear, pitting fault gear, wear fault gear and broken tooth fault gear is simulated, and then the representative curves are created to compare the simulation data of normal driving wheel and fault driven wheel and the predicted value of twins in a meshing process. Finally, the relevant parameters are calculated to compare the off-line data of finite element simulation with the system prediction data.

**Figure 10:**
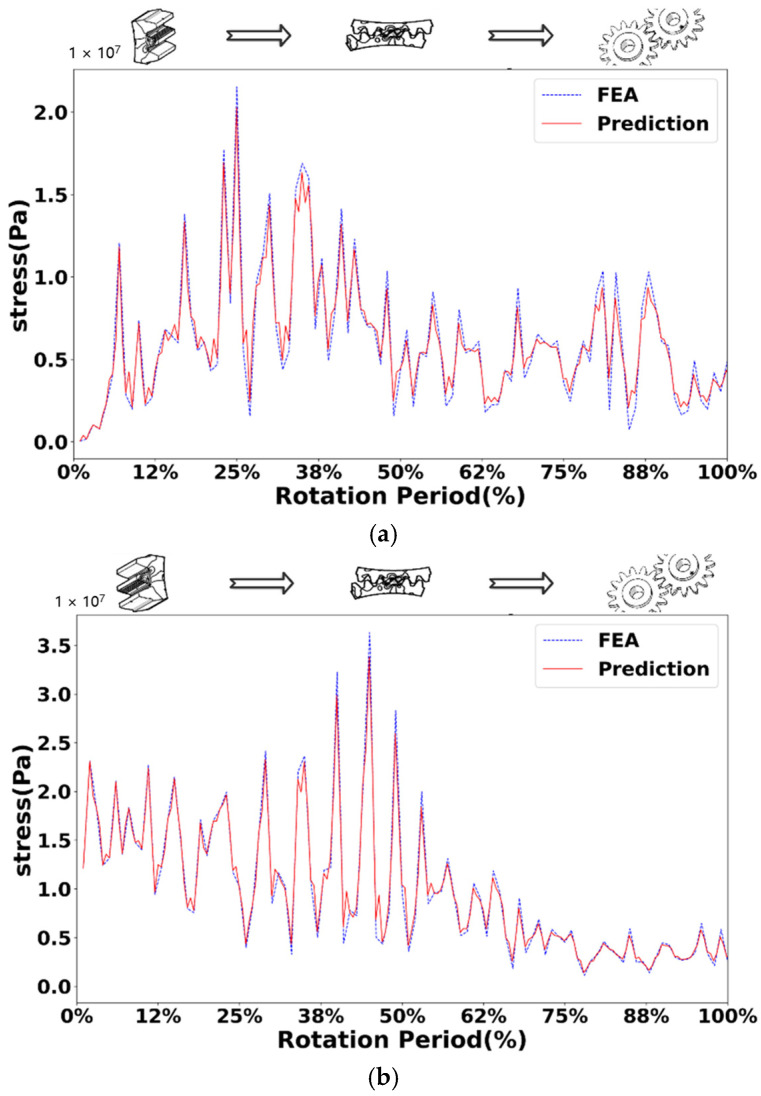
Comparison of Stress Predictions for Normal Gears. (**a**) Comparison of the True and Predicted Stress Values for the Driving Gear. (**b**) Comparison of the True and Predicted Stress Values for the Driven Gear.

**Figure 11:**
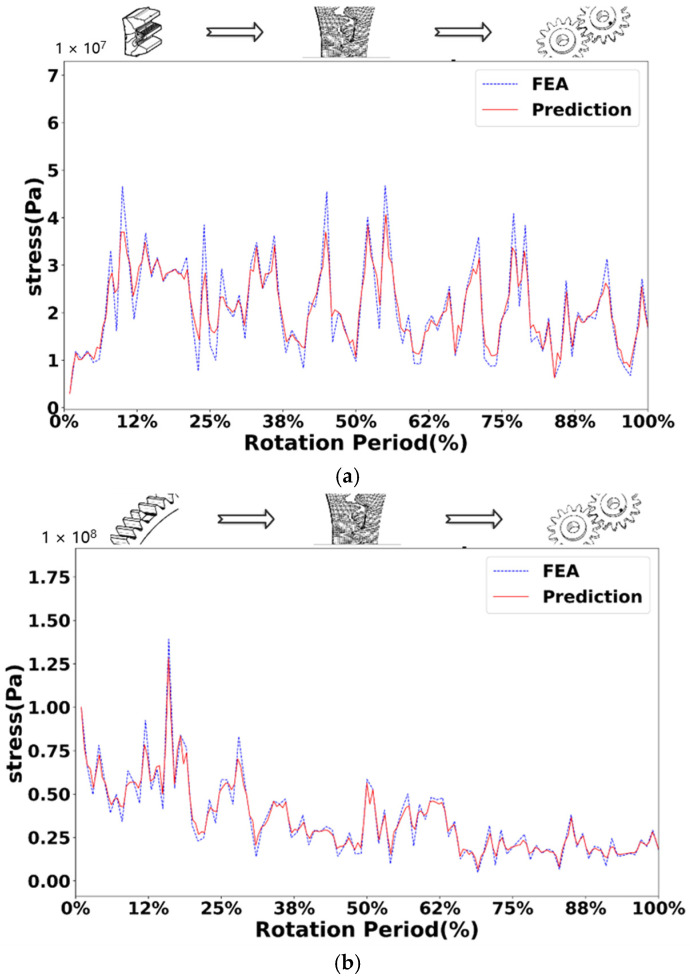
Comparison of Stress Predictions for Gears with Broken Teeth. (**a**) Comparison of the True and Predicted Stress Values for the Driving Gear. (**b**) Comparison of the True and Predicted Stress Values for the Driven Gear.

**Figure 12:**
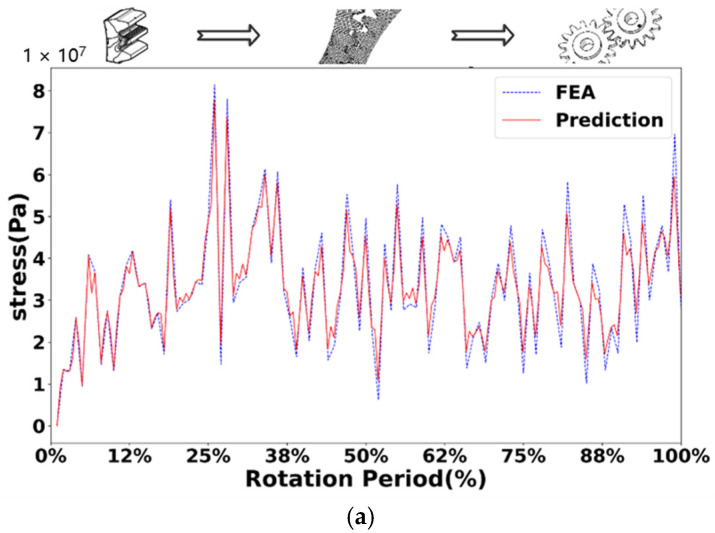

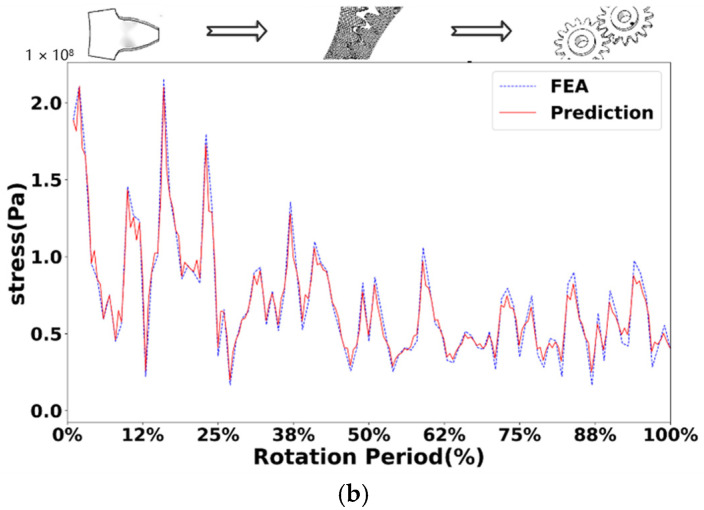
Comparison of Stress Predictions for Worn Gears. (**a**) Comparison of the True and Predicted Stress Values for the Driving Gear. (**b**) Comparison of the True and Predicted Stress Values for the Driven Gear.

**Figure 13:**
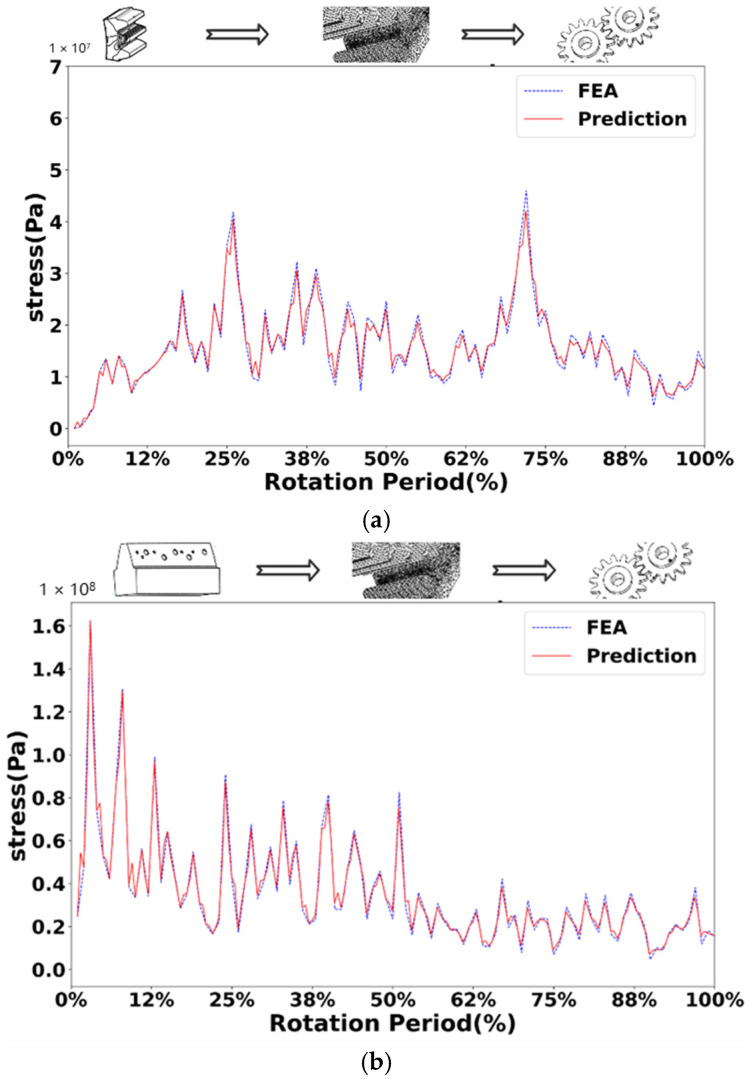
Comparison of Stress Predictions for Pitted Gears. (**a**) Comparison of the True and Predicted Stress Values for the Driving Gear. (**b**) Comparison of the True and Predicted Stress Values for the Driven Gear.

Figure 10, Figure 11, Figure 12 and Figure 13 show the comparison of stress values at observation points for the normal driving and driven gears during gear meshing in four scenarios. Each figure displays two curves: one represents the data obtained from offline finite element simulation, and the other represents the data collected by the digital twin system under the four conditions. The blue dashed line indicates the stress values predicted by the digital twin model, while the red solid line represents the offline finite element simulation data. From the curves, it can be observed that during gear meshing, the stress on the driving gear remains relatively stable due to its normal operation, though there may be brief, minor stress spikes due to certain factors. In contrast, the stress on the driven gear can experience abrupt changes due to different faults during meshing, potentially leading to stress peaks.

Compared with the normal gear, when the gear fails, the stress of both the driving wheel and the driven wheel in the meshing process will increase. In the meshing process of the driving wheel, its stress is more stable than that of the driven wheel. In the driving wheel of normal gear (as shown in Figure 10a), although the stress will surge briefly, the increase is small, and the overall stress fluctuates around 10 MPa. In the driving wheel of the broken tooth gear (as shown in Figure 11a), the stress changes in a zigzag shape, and the stress fluctuates up and down at 25 MPa, with a large fluctuation range, and the maximum stress is close to 50 MPa. For worn gears, the overall effect is similar to that of normal gears because the teeth have no serious defects. The stress variation trend of the driving wheel of the worn gear (as shown in Figure 12a) is similar to that of the normal gear, but the stress value increases as a whole. However, due to the change of the meshing point of the driven wheel after wear, the overall stress value changes greatly, and fluctuates around 40 MPa as a whole. For the pitting fault, because the distribution of pitting holes on the mating surface is relatively random, the stress near the pitting holes often changes greatly when the pitting fault is engaged. Although the stress of driving wheel of pitting gear (as shown in Figure 13a) changes around 20 MPa, the stress surge is frequent and large, and the maximum stress reaches 50 MPa.

In the process of gear meshing, due to the existence of faults, the stress of the driven wheel changes obviously and is usually larger than that of the driving wheel. For the driven wheel of normal gear (as shown in Figure 10a), its stress is relatively stable during meshing. Although the stress increases significantly during meshing, the gear has no fault, the increase is small, and the overall operation is relatively stable. However, for the driven wheel with broken tooth fault (as shown in Figure 11a), the stress increased significantly from the beginning, and the maximum stress reached 140 MPa. Due to the broken tooth fault, the broken tooth at one end is 10 mm, and the residual tooth will produce a great stress during meshing, but after meshing, the stress of the gear gradually stabilized at 30 MPa. For the driven wheel with wear failure (as shown in Figure 12a), the maximum stress surged to 210 MPa, but the stress after meshing was stable and similar to the stress of the driving wheel, floating up and down at 40 MPa. However, the surge of stress can not be ignored. For the driven wheel with pitting failure (as shown in Figure 13a), due to the existence of pitting holes on the meshing surface, the maximum stress reached 160 MPa, but the duration was short, and it soon recovered to a stable state similar to the driving wheel.

Through the study of four types of gear meshing, and after comparing the prediction results of the digital twin models of driving and driven gears with the finite element simulation curves, it was found that there are certain errors between the predicted values and the finite element simulation data for various types. There exists a specific relationship between the driving and driven gears for each type of gear. For normal gears, there is a clear causal relationship in the timing of stress surges. Specifically, after the stress surge in the driving gear, the stress value in the driven gear also rapidly increases shortly thereafter. However, for the three types of faulty gears, there is no apparent sequence in the timing of stress surges. In addition to the stress surge that occurs during smooth operation until meshing contact, the stress surge phase in the driving gear can also increase due to the asymmetry of the fault defect. For example, the stress surge in the driving gear with broken teeth is more frequent, the driving gear with worn teeth also exhibits a significant stress increase at the end of the meshing phase, while pitting corrosion shows two distinct stress surges. Furthermore, compared to the driven gears of normal gears, the stress surge locations in the driven gears of faulty gears occur significantly earlier, with larger magnitudes of numerical surges. The stress values in the driven gears of the three types of faulty gears are significantly higher than those of normal gears, and their stress values after smooth operation following meshing are similar.

When comparing the meshing curves of different types of gears, a variety of parameters can be used. Among them, mean square error (MSE) and coefficient of certainty (R2) are commonly used regression analysis indicators, which are used to evaluate the performance of regression model and the accuracy of prediction. A small MSE value means that the average difference between the predicted value and the real value of the model is small, which indicates that the prediction accuracy of the model is high. The value of R2 is between 0 and 1, and the closer the value is to 1, the stronger the ability of the model to explain the variability of dependent variables, and the better the fitting effect between the predicted value and the real value. Specifically, MSE provides a quantitative value to measure the prediction error of the model; R2 provides a percentage, reflecting the ability of the regression model in explaining the variability of dependent variables.

Specifically, R2 represents the variability between the real value and the predicted value of the test point. The closer its value is to 1, the higher the fitting degree between the predicted value and the real value is. In this verification, when R2 is greater than 0.85, it is determined that the predicted value and the real value have good fitting regression effect. MSE needs to square the difference between the predicted value and the real value of each test point, and then calculate its average value. A smaller MSE value can better illustrate the high prediction accuracy of the model. In contrast, the value of R2 is limited within a range, which is more convenient for comparison between different prediction models, while MSE does not limit the range, and the gap between different models is too large. In contrast, R2 is selected in this paper. Table 3 shows the R2 value of the stress prediction model of driving wheel and driven wheel.

It can be seen from Table 3 that under different working conditions such as normal gear, broken gear, worn gear and pitted gear, the determination coefficient (R2) of the stress prediction model of driving wheel and driven wheel is greater than 0.85, and the minimum value is also greater than 0.92, indicating that the prediction model has good fitting performance. Specifically, the R2 value of the driven gear is the highest under the condition of worn gear, reaching 0.9634, indicating that the prediction accuracy of the stress response of the model is the best under such fault conditions. The overall comparison shows that the R2 value of the driven wheel is generally higher than that of the driving wheel in all kinds of working conditions, indicating that the model has stronger stability and generalization ability in the stress prediction of the driven wheel. Further statistics show that the average R2 value of the driving wheel stress prediction is 0.9324 and that of the driven wheel is 0.9530, both of which are at a high level, which verifies the feasibility and effectiveness of the digital twin prediction model under a variety of typical fault conditions. Although there are some error fluctuations, the overall prediction performance meets the accuracy requirements.

### 4.2. Fault Diagnosis Algorithm Based on Wavelet Transform and Deep Residual Networks

#### 4.2.1. Wavelet Transform

The theory of wavelet transform was developed by French theoretical mathematician Grossmann and French mathematician Morlet, among others. It has shown excellent applications in fields such as signal analysis and image processing [20]. The Fourier Transform (FT) has certain limitations in time-domain signal analysis. To address this issue, Dennis Gabor introduced the window function and proposed Short-Time Fourier Transform (STFT). STFT performs segmented analysis of the signal using a fixed window function, enabling time-frequency analysis [21]. However, the instability of the window function can lead to errors in frequency-domain analysis. Both narrow and wide window functions can affect the precision of time-domain signal characteristics. Wavelet Transform employs a novel approach by replacing the infinite-length sinusoidal basis functions in FT with finite-length and decaying wavelet bases. Compared to STFT, wavelet transform incorporates translation factors and scales in local analysis, allowing the resolution of the window function to be adaptively adjusted according to the characteristics of the frequency. This results in superior time-frequency analysis by capturing the local characteristics of the signal in both time and frequency domains.

In this paper, continuous wavelet transform is used to convert the raw vibration signals of gear transmissions into two-dimensional time-frequency images. Let $\xi \left(t\right)\epsilon L^{2}\left(R\right)$ be a function. If the function $\xi \left(t\right)\epsilon L^{2}\left(R\right)$ satisfies the condition of constant resolution after being transformed by Fourier transform to $\hat{\xi }\left(\bar{\omega }\right)$, the condition is given by:

(11) $$C_{\xi }=\underset{R}{\int }\frac{{\left|\hat{\xi }\left(\bar{\omega }\right)\right|}^{2}}{\left|\omega \right|}d\omega <\infty$$

Then, the function $\xi \left(t\right)$ is considered the mother wavelet or basic wavelet. By scaling and translating the basic wavelet function $\xi \left(t\right)$, a sequence of wavelets is obtained:

(12) $$\xi_{ab}\left(t\right)=\frac{1}{\sqrt{\left|a\right|}}\xi \left(\frac{t-b}{a}\right)\;\;\;\;\;\;\;\;\;a,b\epsilon R;a\neq 0$$

where a and b represent the scaling and translation parameters, respectively.

For any signal $\beta \left(t\right)$, the continuous wavelet transform is given by:

(13) $$W_{\beta }\left(a,b\right)=\beta ,\xi_{ab}={\left|a\right|}^{-\frac{1}{2}}\underset{R}{\int }\beta \left(t\right)\bar{\xi \left(\frac{t-b}{a}\right)}dt$$

The reconstruction formula for the continuous wavelet transform is given by:

(14) $$\beta \left(t\right)=\frac{1}{C_{\xi }}\int_{-\infty }^{\infty }\int_{-\infty }^{\infty }\frac{1}{a^{2}}W_{\beta }\left(a,b\right)\beta \left(\frac{t-b}{a}\right)dadb$$

In the analysis process, the wavelet sequence $\xi_{ab}\left(t\right)$ generated by the transformation of the basic wavelet $\xi \left(t\right)$ acts as an observation window. This ensures that the basic wavelet function $\xi \left(t\right)$ satisfies the following conditions:

(15) $$\int_{-\infty }^{\infty }\left|\xi \left(t\right)\right|dt<\infty$$

Considering that the function $\hat{\xi }\left(\bar{\omega }\right)$ after the Fourier transform is also continuous, it must satisfy the condition that its value is zero at the origin.

(16) $$\hat{\xi }\left(0\right)=\int_{-\infty }^{\infty }\xi \left(t\right)dt=0$$

At the same time, the stability of the results must meet the following conditions:

(17) $$A\leq \overset{\infty }{\underset{-\infty }{\sum }}{\left|\hat{\xi }\left(2^{-j}\omega \right)\right|}^{2}\leq B\;\;\;\;\;\;\;\;\;0\leq A\leq B\leq \infty$$

#### 4.2.2. Deep Residual Networks

(1) Convolutional Neural Networks

**Figure 14:**
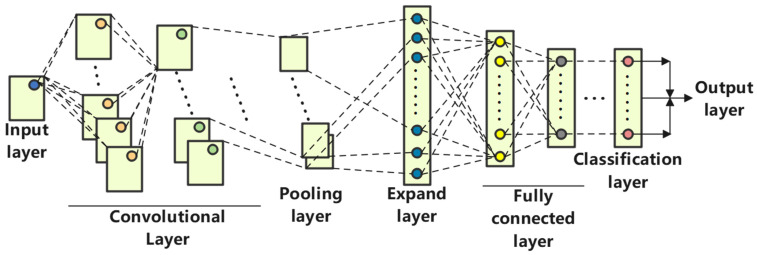
Structure Diagram of a Convolutional Neural Network.

Figure 14 illustrates the structure of a Convolutional Neural Network (CNN). CNNs are widely used in fields such as image processing and audio recognition, and they are built upon convolutional operations and deep learning principles. In a CNN, each layer consists of multiple sets of two-dimensional networks, with each network comprising several independent neurons, adjacent neurons, and neurons that operate independently from one another. Sharing weights within the CNN structure effectively reduces the number of connections and weight settings between network layers, thereby enhancing the learning efficiency of the network model. Besides the input and output layers, a CNN includes convolutional layers, pooling layers, flattening layers, fully connected layers, and classification layers [22].

The input layer (Input) processes multi-dimensional data in a standardized form (mean subtraction, normalization, PCA).

In the convolution layer (Convolution), the convolution kernels perform convolution operations on the output of the previous layer. The output of each layer is the result of the convolution computation with the kernels, as represented by the mathematical model below [23]:

(18) $$\rho_{i}^{l+1}\left(j\right)=M_{i}^{l}*x^{l}\left(j\right)+a_{i}^{l}$$

where $M_{i}^{l}$ is the weight of the i convolution kernel in the l convolution layer;

$a_{i}^{l}$ is the bias of the i convolution kernel in the l convolution layer;

$\rho_{i}^{l+1}\left(j\right)$ is The input to the l+1 convolution layer;

$x^{l}\left(j\right)$ is the j receptive field in the l convolution layer; ∗ is convolution operation.

The activation function used is LeakyReLU. It is applied to construct output features. The LeakyReLU activation function enhances the separability of features by performing nonlinear processing on the convolution outputs. When the input is greater than zero, the function’s slope remains 1, which helps mitigate the issue of gradient vanishing. The function expression for LeakyReLU is as follows:

(19) $$b_{i}^{l+1}\left(j\right)=\beta \left[\rho_{i}^{l+1}\left(j\right)\right]=max\left[0,\rho_{i}^{l+1}\left(j\right)\right]$$

where $b_{i}^{l+1}\left(j\right)$ is the activation value of $\rho_{i}^{l+1}\left(j\right)$.

**Figure 15:**
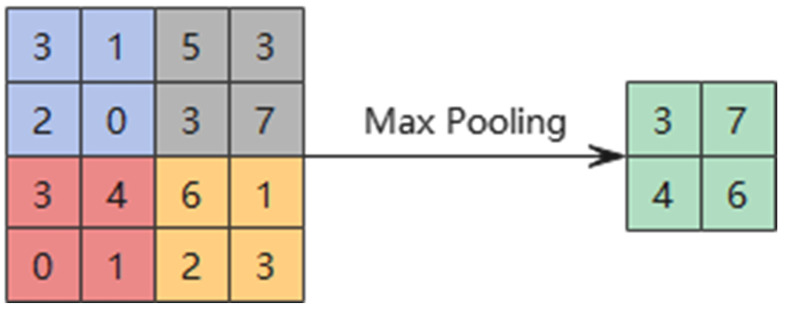
Pooling Principle Diagram.

Figure 15 illustrates the pooling principle. The pooling layer’s objective is to perform dimensionality reduction by dividing the input image into several sub-images and then outputting a single value for each sub-image according to the pooling method. This data down-sampling technique transforms large matrices into smaller ones, thereby reducing computational load and preventing model overfitting.

The maximum pooling technique (MaxPooling) is used in this study, and the formula for maximum pooling is as follows:

(20) $$\sigma_{i}^{l+1}\left(j\right)=max\left[\tau_{i}^{l}\left(t\right)\right]\;\;\;\;\;\;\;\;\left(j-1\right)W+1\leq t\leq jW$$

where $\tau_{i}^{l}\left(t\right)$ is the value of the t neuron in the i feature map of the l layer; W is the width of the pooling region; $\sigma_{i}^{l+1}\left(j\right)$ is the value of the neuron at layer l+1.

The final pooling layer is flattened into a one-dimensional vector. This vector, after the flattening operation, becomes the input to the fully connected layer, which establishes the relationship between the input and output vectors. The formula for the fully connected layer is as follows:

(21) $$\vartheta^{l+1}\left(j\right)=\mu \left[\overset{m}{\underset{i=1}{\sum }}\overset{n}{\underset{t=1}{\sum }}W_{itj}^{l}a_{i}^{l}\left(t\right)+b_{j}^{l}\right]$$

where $W_{itj}^{l}$ is the connection weight between the t neuron in the i feature of the l layer and the j neuron in the l+1 layer; $\vartheta^{l+1}\left(j\right)$ is the logical value of the j neuron in the l+1 layer; μ is activation function ReLu; $a_{i}^{l}\left(t\right)$ is the output value of the t neuron in the i feature of the l layer neuron from the previous layer; $b_{j}^{l}$ is network bias.

The output layer uses a multi-class linear classifier, the SoftMax classifier, for classification. The expression is:

(22) $$I\left(j\right)=SoftMax\left(v^{0}\left(j\right)\right)=\frac{e^{v^{0}\left(j\right)}}{\sum_{k+1}^{M}e^{v^{0}\left(k\right)}}$$

where $e^{v^{0}\left(j\right)}$ is the logic value of the j neuron in the output layer; M is the number of classes.

(2) Deep Residual Shrinkage Network

Deep residual networks alleviate the overfitting problem by introducing residual connections, which allow gradients to propagate directly between layers. This enables the model to be effectively deepened, capturing more complex features. ResNet demonstrates a stronger learning capability when dealing with complex nonlinear relationships, making it particularly suitable for handling high-dimensional and diverse fault data. The residual shrinkage network is an improved version of ResNet, aimed at enhancing the feature learning ability for high-noise vibration signals and the probability of detecting equipment faults using deep learning methods. It consists of residual blocks, soft thresholding, and attention mechanisms. The outputs of each layer in the Deep Residual Shrinkage Network (DRSN) are as follows:

(23) $$I\left(j\right)=SoftMax\left(v^{0}\left(j\right)\right)=\frac{e^{v^{0}\left(j\right)}}{\sum_{k+1}^{M}e^{v^{0}\left(k\right)}}$$

(24) $$b^{\prime }=m^{l+2}LeakyReLU\left(m^{l+1}b^{l}+a^{l+1}\right)+a^{l+2}$$

(25) $$b^{S}=\left\{\begin{array}{l} b^{\prime }-b\;\;\;\;\;\;\;\;\;b^{\prime }>b\\ 0\;\;\;\;\;\;-b\leq b^{\prime }\leq b\\ b^{\prime }+b\;\;\;\;\;\;\;b^{\prime }<-b \end{array}\right.$$

(26) $$b^{l+2}=b^{S}+F\left(b^{l}\right)$$

In which $b^{l}$ is the input size, C×W, the soft threshold of $b^{l+1}$ is obtained from the LeakyReLU function of the hidden layer 1 and serves as the input to the next layer, with the final residual term $F\left(b^{l}\right)$ added to obtain $b^{l+2}$.

The deep residual shrinkage network is composed of a series of residual blocks, each of which can be represented as:

(27) $$yx^{l+1}=x^{l}+F\left(x^{l},M^{l}\right)$$

**Figure 16:**
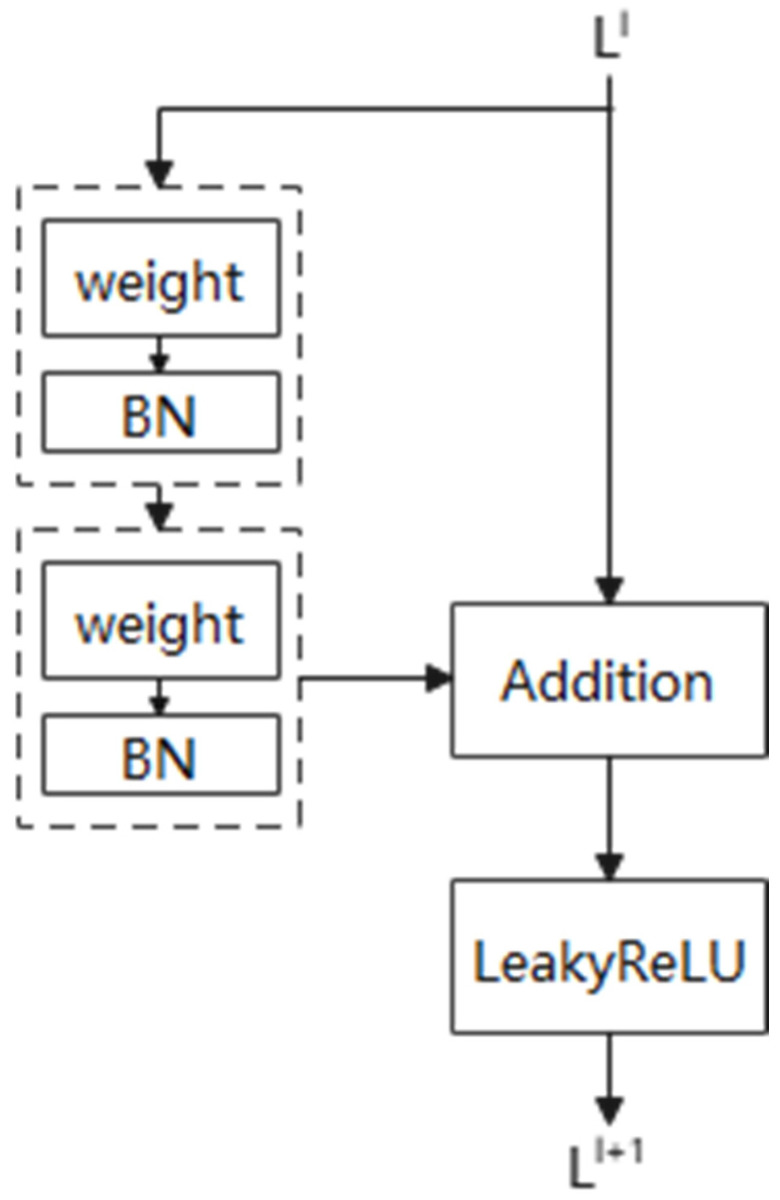
Residual block flowchart.

Figure 16 illustrates the flowchart of a residual block. The residual block is divided into two parts: the direct mapping and the residual. $L^{1}$ represents the direct mapping, while $F\left(L^{1},M^{1}\right)$ denotes the residual part, which typically consists of 2 to 3 convolution steps.

In signal denoising algorithms, the soft-thresholding function is commonly used to set irrelevant data to zero or retain useful data. The specific formula is as follows:

(28) $$y=\left\{\begin{array}{l} x-q\;\;\;\;\;\;\;\;\;\;\;x>q\\ 0\;\;\;\;\;\;\;-q\leq x\leq q\\ x+q\;\;\;\;\;\;\;\;\;x<-q \end{array}\right.$$

where x is input feature; y is output feature; q is threshold.

The derivative of the output data with respect to the input data for the soft thresholding function is:

(29) $$\frac{\partial y}{\partial x}=\left\{\begin{array}{l} 1\;\;\;\;\;\;\;\;\;\;\;\;\;\;\;\;x>q\\ 0\;\;\;\;\;-q\leq x\leq q\\ 1\;\;\;\;\;\;\;\;\;\;\;\;\;x<-q \end{array}\right.$$

Using a specific neural network model, all samples will be trained based on the noise level, automatically setting the threshold. This significantly reduces the reliance on signal processing theories and allows for empirical validation of the method’s effectiveness.

**Figure 17:**
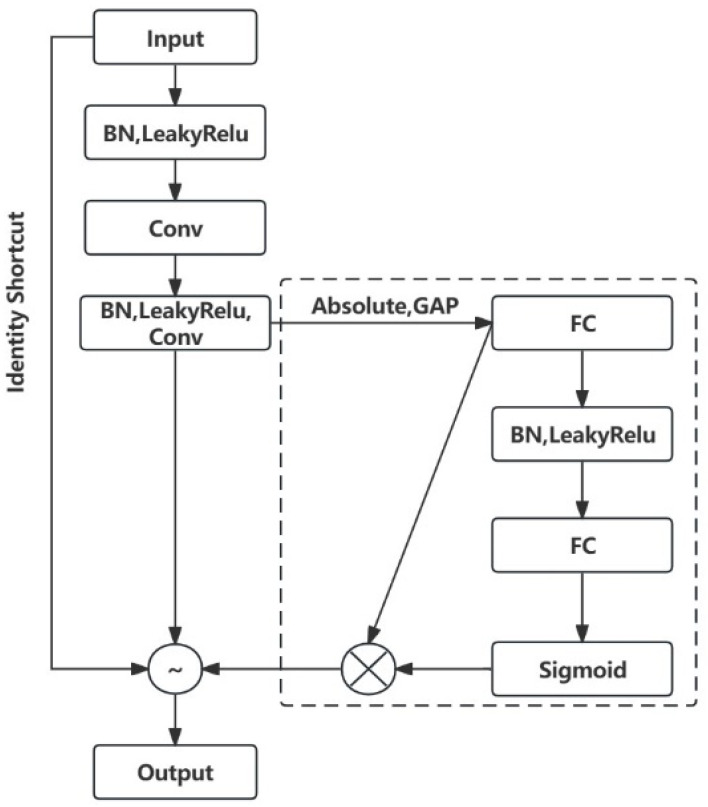
The basic deep residual shrinkage module.

Figure 17 illustrates the basic deep residual shrinkage module. DRSN incorporates an attention mechanism subnetwork and soft thresholding before the output of the residual network. The attention mechanism subnetwork adaptively evaluates the importance of input features to obtain a set of thresholds. Soft thresholding maps input features to output features, setting to zero those components with absolute values below the threshold and shrinking those above the threshold towards zero. This process filters out noise-related features and enhances the network’s robustness to noise and redundant information.

(3) The fault diagnosis method based on wavelet transform and deep residual shrinkage network.

In DRSN, a 64 × 3 × 3 convolutional kernel is used to extract key features from the feature maps. The subsequent four layers of convolutional structures stack basic deep residual shrinkage modules to build the backbone network, which is used for both denoising and feature extraction. After the extracted features are processed through the max-pooling layer, they are flattened and processed in the fully connected layer to extract feature vectors. The feature vectors are then classified through the Softmax layer to obtain the final fault diagnosis results. Details on the number of kernels, strides, sizes, and output dimensions for each layer in the deep residual shrinkage network are provided in Table 4.

To validate the effectiveness of the fault diagnosis model used, this study collected gearbox fault data based on the QPZZ-II test rig for training and validation. The gearbox fault dataset comprises four types, including one normal gear type and three fault gear types. A detailed description of these gear types is provided in Table 5.

After the test rig operated stably, signals were collected using the CA-YD-116T piezoelectric accelerometer. Data were recorded at a rotational speed of 800 r/min under four conditions: normal, broken teeth, pitting, and wear. The collected raw data were subjected to continuous wavelet transform to generate 2D time-frequency maps, which were then used to construct a convolutional neural network (CNN) diagnostic model. There are 1920 time-frequency charts in total. The wavelet time-frequency maps of the gear signals were processed simply before being input into the CNN model for fault analysis. The specific processing steps are as follows:

1. Input the obtained raw signals into the continuous wavelet transform algorithm to generate wavelet time-frequency maps.
2. Remove the axes, labels, and legends from the time-frequency maps to facilitate processing by the neural network.
3. Perform grid-based normalization and compression of the images, setting the pixel dimensions to 224 × 224.

**Figure 18:**
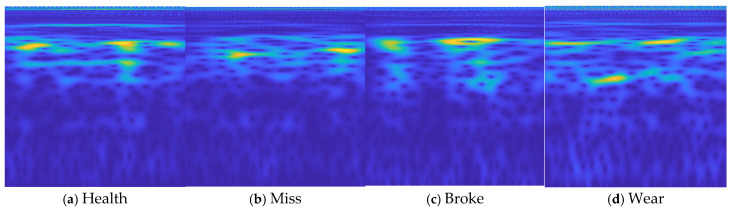
Preprocessed Wavelet Time-Frequency Maps.

Figure 18 shows the preprocessed wavelet time-frequency maps. To train and test the convolutional neural network model, different segments must be extracted from the continuous raw signals, and multiple wavelet time-frequency maps should be generated as training and testing data.

**Figure 19:**
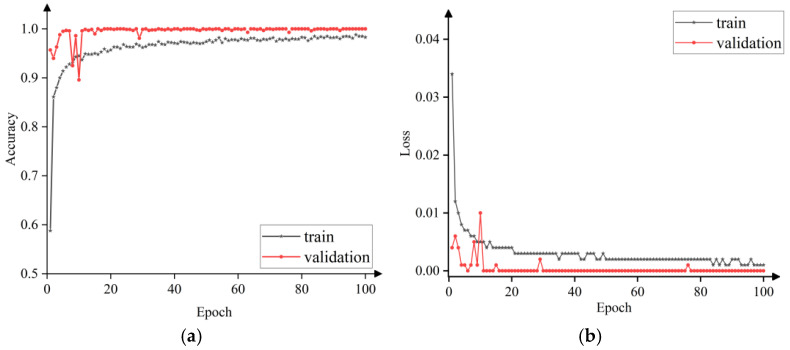
Accuracy and Loss Comparison Curves for Training and Testing Sets of the Fault Diagnosis Model. (**a**) Accuracy Comparison Curves for Training and Testing Sets. (**b**) Loss Comparison Curves for Training and Testing Sets.

The collected signal data is processed by wavelet transform and preprocessing, and the wavelet time-frequency maps of four working conditions are obtained. Among them, 60% of the images are used to train the convolutional neural network, and the remaining images are used to test the accuracy of the model. Figure 19 shows the comparison curve of accuracy and loss rate of fault diagnosis model training set and test set, in which the red line and black line represent the accuracy and loss rate of training set and test set respectively. After 100 iterations, the diagnostic accuracy of the test set reached 99.45%, and the loss rate was 0.002. As can be seen from Figure 19, with the increase of training rounds (epoch), the accuracy of the training set and the verification set gradually increased and tended to be stable. Although the validation set fluctuated at the initial stage of training, the accuracy of both remained at a high level and the subsequent fluctuation was minimal as the training progressed. This shows that the model can maintain a relatively stable performance in multiple runs with indepth training, and finally achieve a high and stable accuracy in both the training set and the verification set, reflecting that the model has good stability in the overall training process, can maintain performance consistency in different rounds of training, and does not have a significant performance shock, showing that the model has good stability in multiple runs.

To verify the superiority of the proposed method, the accuracy, accuracy, recall rate and F1 score were used to comprehensively analyze and evaluate the model

(30) $$\mathrm{Accuracy}=\frac{TP+TN}{TP+TN+FP+FN}$$

(31) $$\mathrm{Precision}=\frac{TP}{TP+FP}$$

(32) $$\mathrm{Recall}=\frac{TP}{TP+FN}$$

(33) $$F_{1-score}=\frac{2\times Precision\times Recall}{Precision+Recall}$$

where *TP* is the number of samples correctly predicted as a positive class; *TN* is the number of samples correctly predicted to be negative; *FP* is the number of negative samples that are incorrectly predicted as positive; *FN* is the number of positive samples that are incorrectly predicted as negative.

**Figure 20:**
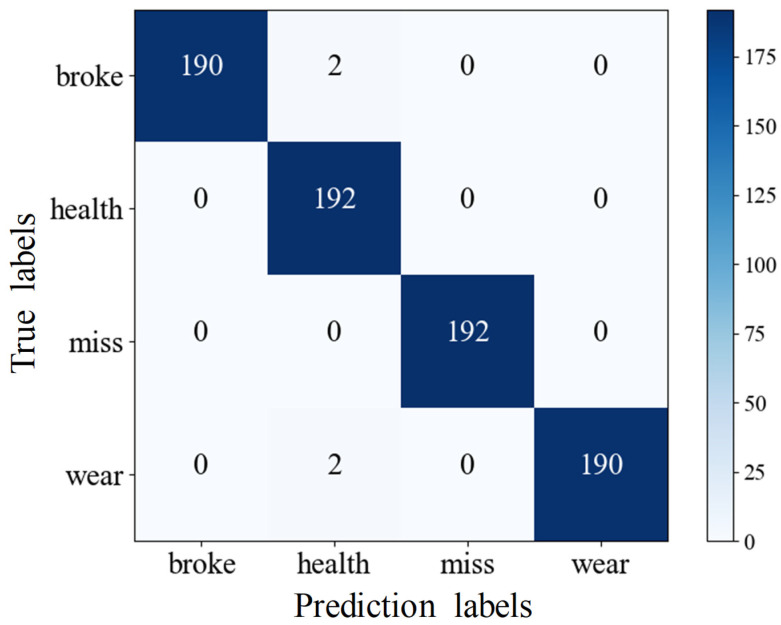
Confusion Matrix.

Figure 20 shows the confusion matrix for the fault diagnosis model, which assesses the accuracy of the deep residual network in classification tasks. The rows represent the predicted labels—pitting, healthy, broken teeth, and wear—while the columns represent the actual labels. The numbers on the diagonal indicate the number of correct predictions for each class, and off-diagonal numbers show the count of incorrect predictions. Higher values on the diagonal signify better classification accuracy. The results reveal that the detection rates for pitting, broken teeth, and wear are all 100%, while the accuracy for healthy gears is 99%.

**Figure 21:**
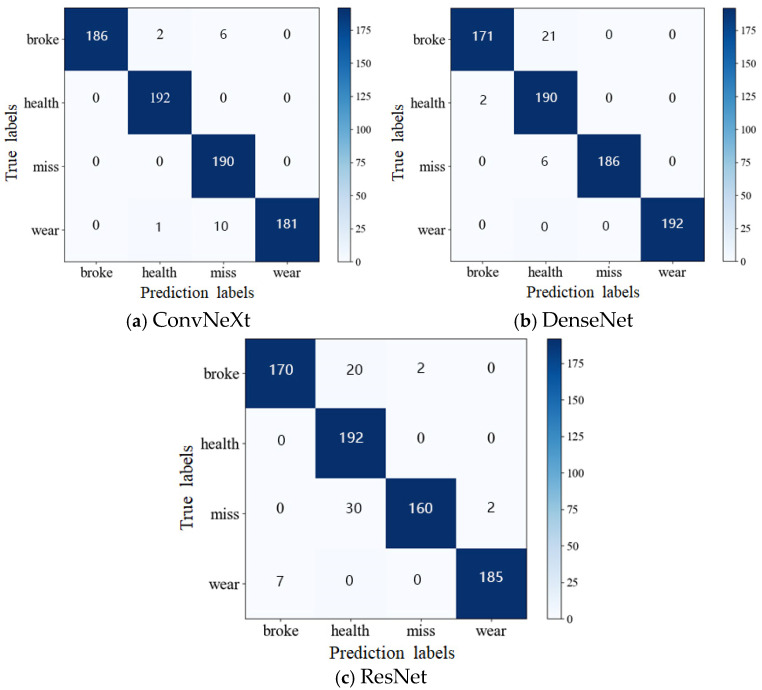
Confusion matrices for different models.

Popular deep learning models such as ResNet, DenseNet, and ConvNeXt were selected for comparison. Both DenseNet, ConvNeXt, and the method proposed in this manuscript are improvements based on ResNet. By comparing them with ResNet and the other two methods, the superiority of the proposed approach is validated. The Adam optimizer was used with a learning rate of 0.0001, and the loss function was the cross-entropy loss function. The models were built and trained using PyCharm on an NVIDIA 4070 GPU. Training accuracy and other evaluation indicators are shown in the Table 6:

After the model testing was completed, confusion matrices for the three models were plotted, as shown in Figure 21. Compared to these models, the proposed model exhibited the fewest misclassifications. Specifically, ConvNeXt had 19 misclassifications, DenseNet had 29, and ResNet had 61. The lower accuracy of ConvNeXt and DenseNet can be attributed to their deeper architectures, leading to slower convergence during iterations. Meanwhile, ResNet, being relatively simpler, failed to fully extract fault features. By comparing the proposed model with these three models, its superiority was validated.

**Figure 22:**
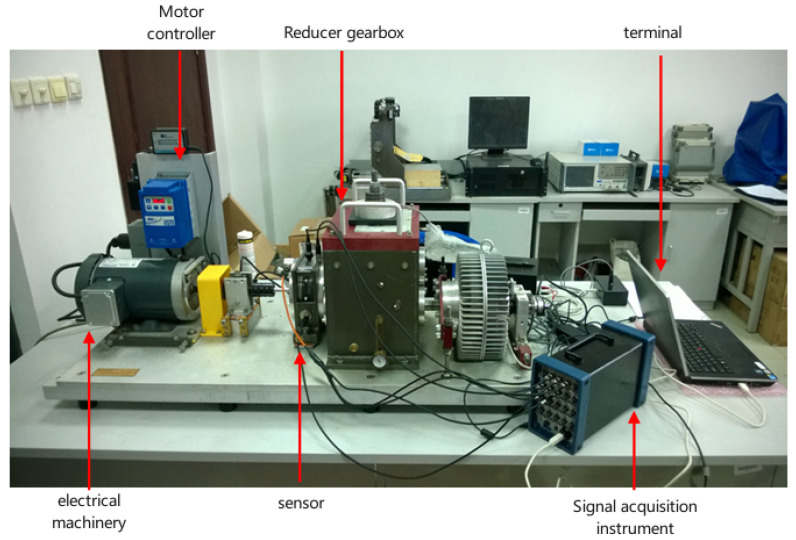
Power transmission simulation test bench.

In order to verify the applicability of the proposed diagnostic model, a power transmission simulation test bench was built, as shown in Figure 22. It is mainly composed of motor, motor controller, reduction gearbox and other devices. The acceleration sensors are installed on the vertical, horizontal and axial measuring points on the top of the gearbox, with a sampling frequency of 12,800 Hz, a rotating speed of 2400 r/min and a load of zero to collect the vibration signals of normal and different fault types (broken teeth, pitting corrosion and wear).

**Figure 23:**
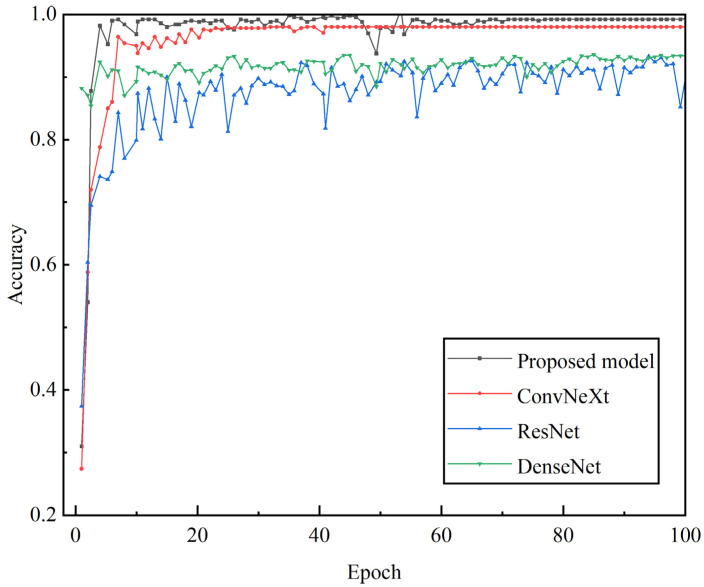
Comparison curve of test set accuracy.

In order to prove the superiority of the proposed model in gearbox fault diagnosis, it is compared with RESNET, densenet and convnext models. The training parameters of each model are consistent in the experiment. Figure 23 shows the iterative trend of the accuracy of the test set. It can be seen that compared with the other three network models, the proposed model shows certain advantages. During the training process, the accuracy of the curve of the proposed model increased rapidly and remained stable after reaching a high level, and there was almost no significant fluctuation in the later stage. Compared with other models (such as RESNET curve has more fluctuations, densenet also has some fluctuations), this model can maintain a stable high accuracy rate in the late training period without significant performance fluctuations, which reflects its good stability, can maintain a robust performance output in different training rounds, and the model performs well in the diagnosis accuracy of normal and three fault types, with an accuracy rate of 99.48%.

## 5. Development of an Integrated Form-Performance Gearbox Control System Platform

### 5.1. Platform Development

**Figure 24:**
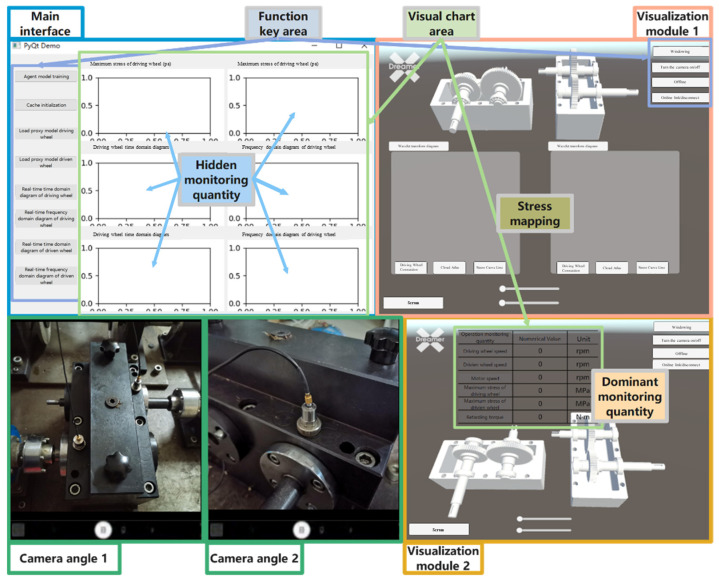
Interface of the Integrated Gearbox Status Monitoring System.

Figure 24 shows the interface of the integrated gearbox status monitoring system. Based on the framework of the integrated gearbox monitoring system, it employs virtual-physical mapping and data interaction among the physical space, virtual twin space, and associated twin data. This is combined with the design of functional modules, system processes, and the integrated gearbox monitoring system platform.

As shown in Figure 24, the integrated gearbox status monitoring system consists of three main modules. The first module is the system visualization main interface, designed using Qt-designer in Python. This interface includes a functional button area with switches for virtual-physical interaction, performance prediction model, fault signal monitoring, data initialization, and model training, and a functional operation area for data preprocessing and model training during proxy model training, as well as real-time monitoring of the maximum stress on the driving and driven wheels and the time-frequency diagrams of the original signals. The second module involves 3D model presentation, where pressing the “Load Proxy Model Driving Wheel—Driving Wheel Connection—Cloud Chart” buttons sequentially presents the 3D models in the physical entity and virtual twin windows. The virtual world, constructed using Unity 3D, mainly includes the gearbox virtual twin workspace and function button area, featuring sliders for gear meshing angle and speed, and buttons for stress cloud diagrams of driving and driven wheels, wavelet transform diagrams, maximum stress, interface curve windowing, offline inspection, and emergency stop. The third module, Visualization Module 2, displays relevant data for the physical entity and the virtual twin, such as gear and motor speed, braking torque, and maximum stress.

### 5.2. Data Fusion and Visualization

**Figure 25:**
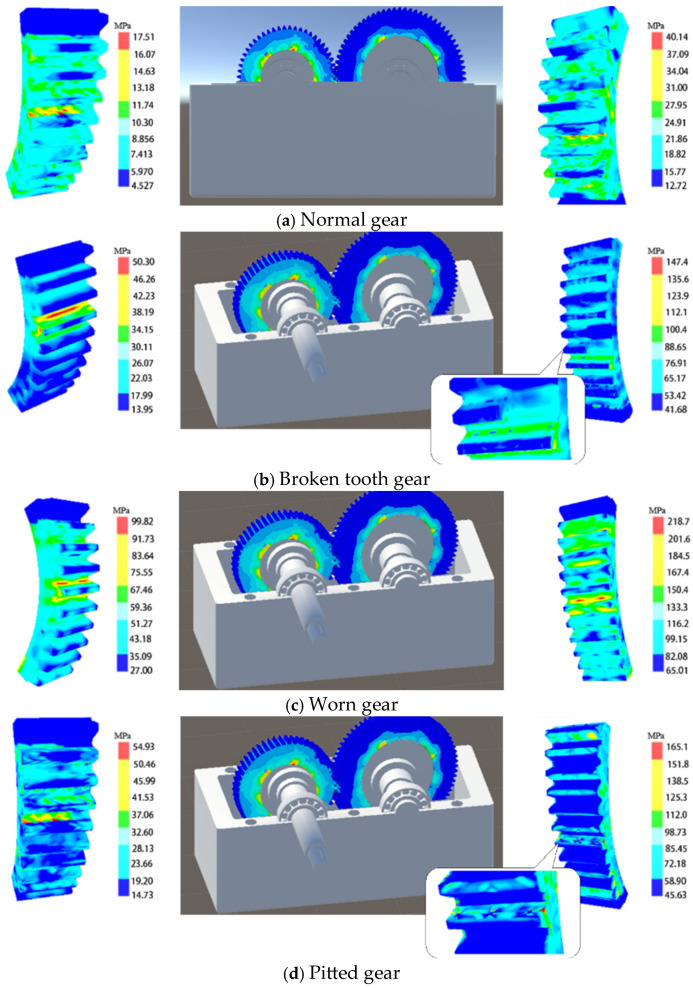
Visualization of Stress Cloud Maps for Virtual Twin Gears in Different Conditions.

By utilizing computer graphics technology, the digital twin system’s visualization is realized within the integrated gearbox monitoring platform, providing visual feedback on gear fault types and mechanical performance. When a fault occurs during gear meshing, the current mechanical performance (Mises stress) of the gear is displayed in real-time using a rainbow color spectrum on a stress cloud diagram. Different stress values are represented by different colors. Figure 25 shows the visualization effect of the stress cloud diagram for the virtual twin gears under various conditions (normal, broken tooth, worn, and pitted). The twin driving gear is located on the left side, while the twin driven gear is on the right. The Mises stress values corresponding to various colors can be referenced from the color threshold table. The highest Mises stress is indicated in red, while the lowest is shown in blue. In the virtual models of normal and worn gears, most of the stress values are represented in green, indicating that the gear meshing impacts a large area and that the entire gear is affected. In contrast, the virtual models of broken tooth and pitted faults are mostly blue, indicating lower values. The meshing positions in Figure 25 are colored red and yellow, indicating the maximum stress at these positions, concentrated at the gear roots, which aligns with the finite element simulation results in Chapter 3. By combining the stress cloud diagram’s rainbow spectrum with its threshold table, the gear fault stress values and their distribution can be clearly observed, enabling timely machine shutdowns to prevent property damage and achieve real-time fault monitoring and high-fidelity dynamic visualization of the gears.

The aforementioned phenomena indicate that the fault condition of the gear affects the forces experienced by both the driving and driven gears. Specifically, when a gear tooth breaks, the remaining teeth bear the entire meshing stress of the gear, but due to their relatively smooth surfaces, the increase in stress is minimal. For pitted gears, the presence of pitting holes on the contact surface reduces the force-bearing area near the holes, leading to a sharp increase in stress. In the case of worn gears, the meshing position shifts compared to a normal gear, which is the primary cause of the most significant increase in stress.

## 6. Experimental Case

### 6.1. Gearbox Data Collection and Data Communication

(1) Sensor Distribution

**Figure 26:**
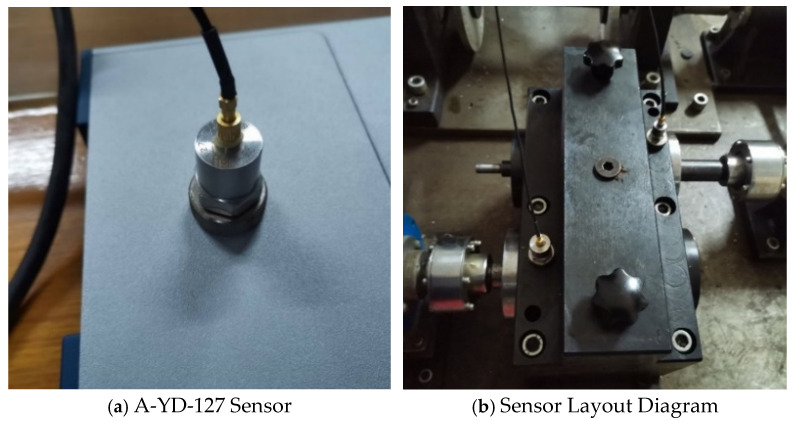
Sensor Placement.

The vibration sensors consist of components such as dampers, springs, and mass blocks. They use the mass block to establish a coordinate system in inertial space to detect the vibration acceleration of the research object relative to the ground or inertial space. The sensors are equipped with transducing components that convert vibration into clear electrical signals. In this experiment, a unidirectional piezoelectric accelerometer sensor model CA-YD-127 is used, which is connected to a serial data acquisition device. Figure 26 illustrates the sensor layout. Since the gears inside the gearbox cannot be accessed directly for signal acquisition, indirect contact methods are employed. Sensors are placed on the end caps of both the input end of the driving gear and the output end of the driven gear.

The experiment was carried out under the stable operation condition of the gearbox, the sampling frequency was set at 5120 Hz, and the sampling duration was 60 s. In order to construct the sample data set, the step size is 150, and each image is generated by 1024 points. Finally, 1920 two-dimensional wavelet time-frequency images are generated as the input samples of the fault diagnosis model for training and verification. The experimental tests were carried out at room temperature in the laboratory, and the temperature was maintained at about 23 °C. The environmental humidity was not controlled or recorded during the experiment.

(2) Data Communication between Physical Entities and Twin Models

The data from physical entities is collected using various sensors tailored to the vibration frequencies of the targets. For the high-frequency vibrations in gearboxes, accelerometers are used to capture real-time vibration signals. The accelerometer data is transmitted to a data acquisition system via serial communication, and then integrated into Unity3D through C# scripts. This setup allows for accurate real-time monitoring and analysis of the gear performance and condition within the digital twin model.

The data transmission form for serial communication is as follows: Frame header + Frame length + Data bits + Checksum + Frame tail.

The communication process involves data reception, data verification, data parsing, and repeated execution. The data will be transmitted in ASCII code format. Data reception is handled in Unity3D, which requires the use of the System.IO.Ports communication interface for serial communication. This includes the SerialPort class, which provides control over serial ports, access to I/O, access to serial port driver properties, and port and interrupt status management.

**Figure 27:**
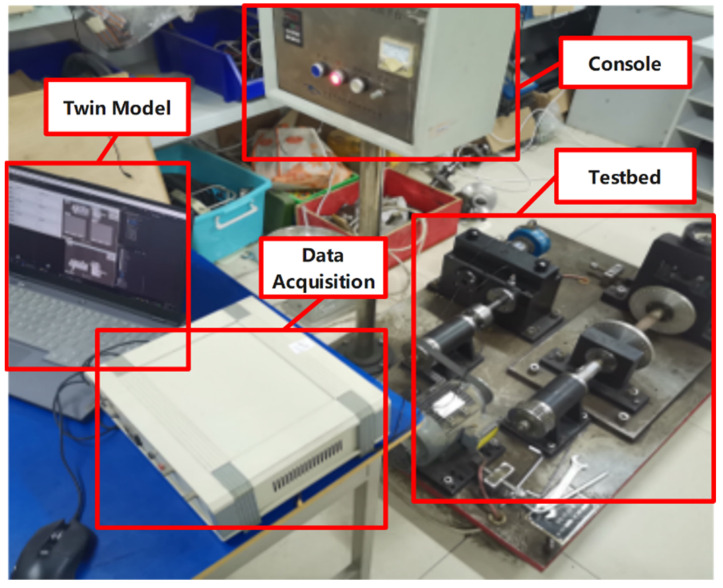
The shape-physical integration gearbox monitoring system test rig.

Connect the QPZZ-II test rig, control console, piezoelectric accelerometers, digital twin model, and data acquisition system for experimental validation. Figure 27 shows the constructed shape-physical integration gearbox monitoring system test rig. This test platform includes the control system (test rig control console), data acquisition system (sensors and data acquisition card), physical machine (QPZZ-II test rig), and the shape-physical integration gearbox monitoring platform (digital twin model).

**Figure 28:**
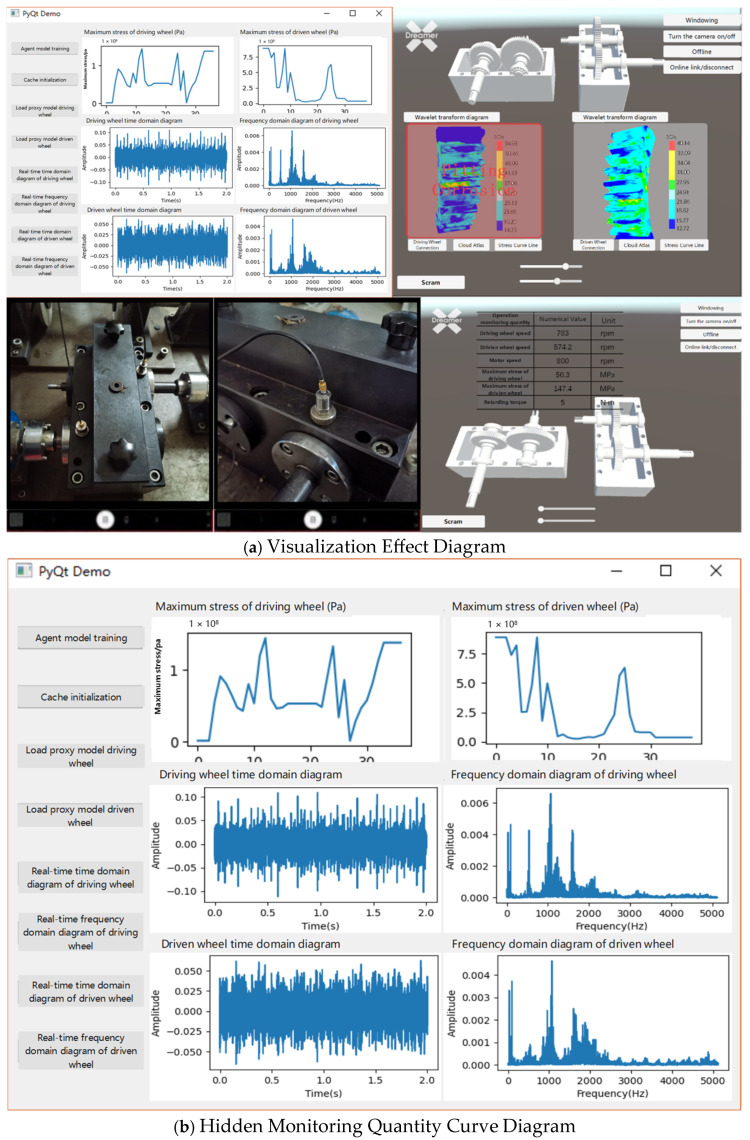
Visualization Effect of Shape-Physical Integration Gearbox Monitoring System.

The virtual twin model collects piezoelectric accelerometer data in real-time and maps it to the 3D model through the fault diagnosis model. Serial communication inputs the rotational speed into the virtual twin model, which, in conjunction with the performance prediction model, maps stress data in real-time onto the color cloud map. This enables real-time visualization of the gearbox gear’s operational performance. The real-time stress cloud map, fault model, maximum stress, time-frequency curve, and related monitoring parameters of the shape-physical integration gearbox monitoring system are illustrated in Figure 28.

**Figure 29:**
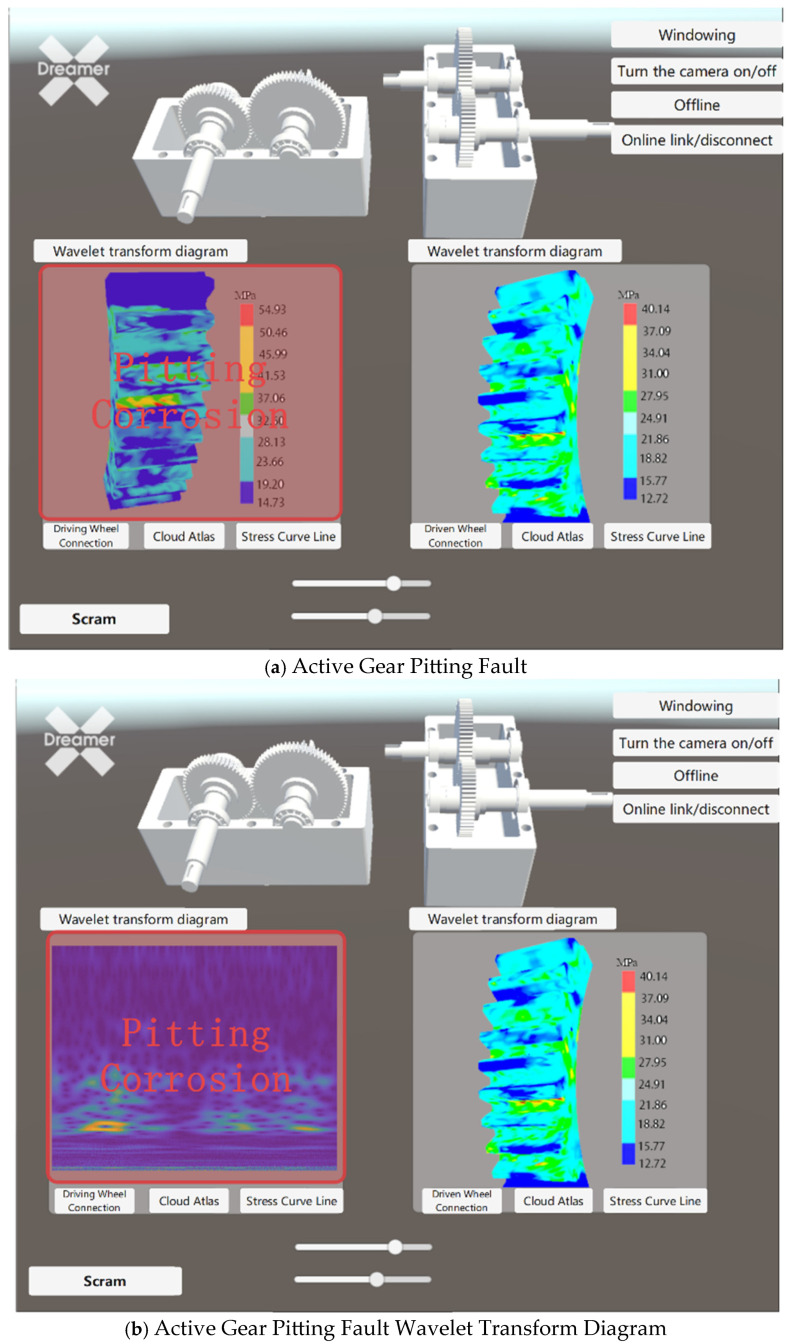
Device Fault Warning Display Effect.

Figure 29 illustrates the device fault warning display effect. To meet various user needs while satisfying design goals, the visualization module has been enhanced with additional features such as wavelet transform diagrams and fault warnings. This allows users to comprehensively monitor the gear operating status. If abnormal vibration signals or faults occur during the operation of the driving or driven gears, the monitoring system will promptly relay the fault information to the monitoring personnel. Upon receiving the fault data, the virtual twin model will display the fault type and issue a warning in the corresponding window. The monitoring personnel can then immediately stop the equipment and perform a thorough inspection upon receiving the alert.

Through testing on the platform, it has been verified that the virtual twin model communicates well with the physical machine and sensors. The virtual twin model is capable of performing fault diagnosis and visualizing the operational performance of the physical machine.

### 6.2. Experimental Verification

**Figure 30:**
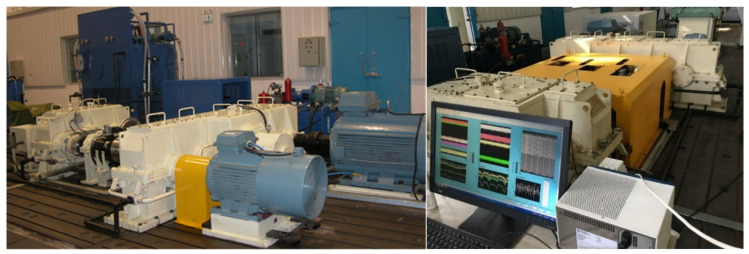
Reducer Test Bench Environment.

Signals from four distinct fault types (normal, tooth breakage, pitting, and wear) were collected on the modified reducer test bench, as illustrated in Figure 30. These signals were input into the established digital twin monitoring system for testing, which includes validation of both fault prediction capabilities and performance characteristic prediction accuracy. While Section 4.2.2, demonstrates the superiority of the Deep Residual Shrinkage Network (DRSN) model in fault recognition, this subsection verifies the holistic predictive capacity of the digital twin system through experimental signal acquisition.

**Figure 31:**
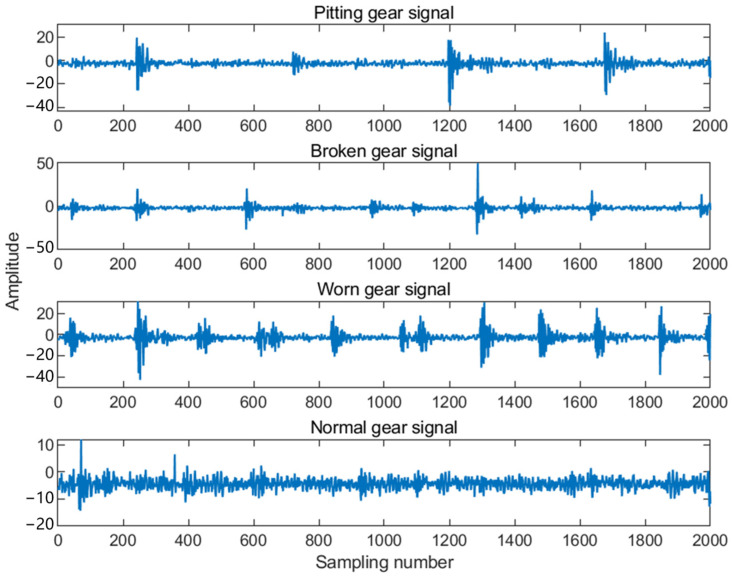
One-dimensional signals of gears with different faults.

The different fault signals collected are shown in Figure 31.

#### 6.2.1. Fault Prediction Accuracy Experiment

**Figure 32:**
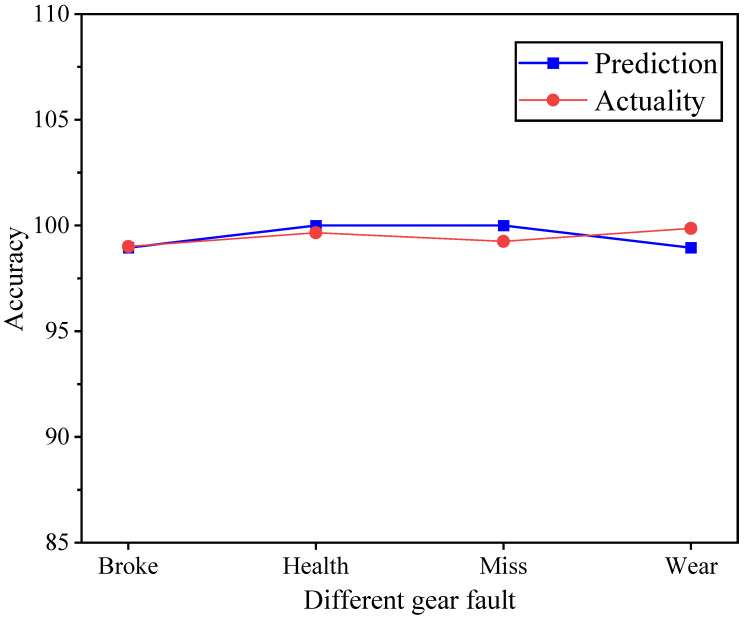
Comparison of experimental and predicted accuracy.

The signals of different faults were input into the digital twin monitoring system for testing, and the final output was the actual prediction accuracy, which was compared with the test data, as shown in Figure 32.

As shown in Figure 32, the accuracy prediction curve of the digital twin monitoring system for the experimental signals closely matches that of the test signals. The predicted accuracy for different faults based on the test signals is: tooth breakage 98.95%, normal 100%, pitting 100%, and wear 98.95%. For the experimental signals, the results are: tooth breakage 99.01%, normal 99.66%, pitting 99.25%, and wear 99.86%. The largest difference, observed in the gear wear fault, is only 0.91%. This demonstrates that the developed digital twin monitoring system has excellent stability and applicability.

#### 6.2.2. Performance Correlation Prediction Experiment

**Figure 33:**
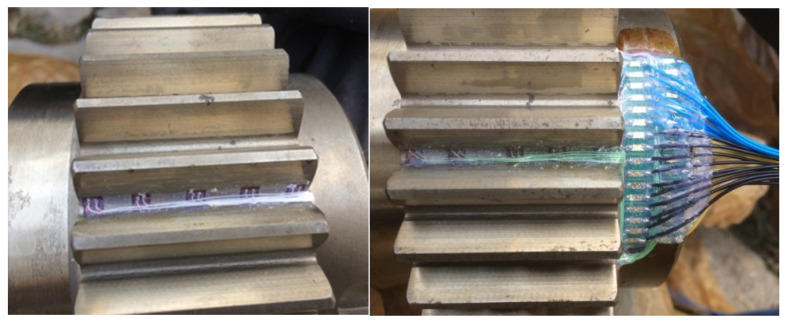
Paste wiring diagram of strain measuring points.

In the performance prediction experiment, strain gauge instrumentation was implemented on the gear teeth to acquire actual stress data, as shown in Figure 33. The figure details the strain gauge mounting and wiring configuration at the gear root zone, where critical stress measurement points were established through precise gauge installation and circuit integration. This instrumentation forms the foundation for obtaining authentic stress data from both the driving and driven gears. The acquired stress signals, collected via an IMC strain data acquisition system, were comparatively analyzed against predictions from the twin model. The coefficient of determination (R^2^) calculated through Equation (34) quantifies the goodness-of-fit between predicted and measured values. The resulting R^2^ values for the driving and driven gear stress prediction models, presented in Table 7, validate the twin model’s predictive accuracy across varying gear fault conditions.

**Figure 34:**
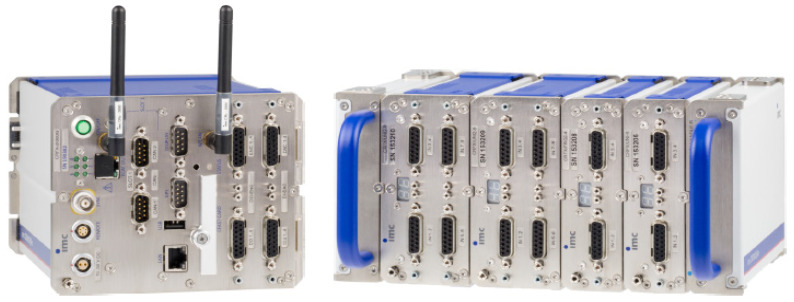
IMC strain collector.

The experimental setup employed an IMC strain data acquisition system (as shown in Figure 34) as the core measurement device. This system features multi-channel synchronous acquisition capabilities, enabling precise capture of stress data from both driving and driven gears under various fault conditions (normal, tooth breakage, wear, and pitting). Its high-precision signal conditioning modules and high-speed sampling characteristics (50 kHz/channel) ensured data reliability, providing fundamental support for subsequent predictive model accuracy analysis.

The coefficient of determination (R^2^) is a commonly used metric in regression analysis that evaluates the performance of a model. The value of R^2^ ranges from 0 to 1; the closer the value is to 1, the stronger the model’s ability to explain the variability of the dependent variable, indicating a better fit between predicted and actual values. Specifically, R^2^ provides a percentage that reflects the model’s capacity to explain the variability of the dependent variable. The use of R^2^ to assess the error between finite element simulations and model predictions can be expressed as:

(34) $$R^{2}=1-\frac{\sum_{i=1}^{n}{\left(y_{i}-\hat{y_{i}}\right)}^{2}}{\sum_{i=1}^{n}{\left(y_{i}-\hat{y}\right)}^{2}}$$

In the equation: $y_{i}$ represents the true value of the test set at the i-th point, ${\hat{y}}_{i}$ represents the predicted value at the i-th point, $\hat{y}$ represents the mean of all true values in the test set, n represents the sample size.

Table 7 presents the R^2^ values of the stress prediction models for the driving wheel and the driven wheel in this experiment.

According to Table 7, the R^2^ values under different fault conditions are all greater than 0.85, with the maximum R^2^ value for the driven wheel under tooth breakage being 0.9645, and the R^2^ values for the driven wheel are generally higher than those for the driving wheel. This indicates that the twin prediction model exhibits high prediction accuracy, with a maximum calculated R^2^ value of 0.9645. The average R^2^ value for the stress of the driving wheel is calculated to be 0.9339, while the average R^2^ value for the stress of the driven wheel is 0.9497. These data suggest that there is a certain degree of error in the artificial intelligence prediction model, with the stress fluctuations of the driven wheel being smaller compared to those of the driving wheel. Overall, however, the accuracy of the twin prediction model is acceptable.

## 7. Conclusions

This paper proposes a method for constructing a gear shape-performance integrated digital twin using artificial intelligence, data analysis, and thermo-fluid-solid coupling simulation modeling. This method integrates fault diagnosis technology, finite element methods, and artificial intelligence (AI) models to perform real-time gear fault diagnosis and predict the mechanical properties of actual gears. First, the accuracy of the finite element analysis (FEA) results is validated by comparing the data with previous experimental results using the established finite element model. Next, the geometric and performance data obtained from the finite element analysis are used to build the AI model. Finally, sensor data is utilized as input to achieve the mapping from physical space to digital space. The real-time predictions during gear operation and offline simulations demonstrate good correlation, proving the accuracy of the digital twin model. Additionally, high-fidelity dynamic visualization and health monitoring of the gears are achieved in real-time.

However, it should be noted that due to the difficulty in obtaining the physical monitoring stress data in the real environment during the gear transmission process, the current research on the digital twin stress data comes from the finite element simulation, so the fidelity of the finite element model determines the accuracy of the stress prediction of the digital twin model. The complexity of finite element analysis is reflected in the joint action of various constraints and material characteristics in the coupling state of heat source, lubricating oil, gearbox shell and gear itself in the process of gear meshing. These parameters have a great impact on the results of finite element simulation. In addition, this study has considered load changes and different fault types as important dimensions of input conditions, and enhanced the adaptability of the model by grouping conditions. However, at present, no systematic quantitative analysis of the disturbance sensitivity of more comprehensive input parameters has been carried out. Therefore, the follow-up research will further introduce methods such as local disturbance test to explore the impact of load, rotation angle, fault type and other variables on the output results of the proxy model, so as to improve the robustness and interpretability of the model under complex working conditions. One of the future research directions is to improve the adaptability and reliability of the system in complex environments by optimizing the simulation modeling accuracy, introducing the verification mechanism of measured stress data, and expanding the working condition parameters of the proxy model; The other direction is to improve the fidelity of twins by collecting more parameters of physical entities, which will have great significance for the development of digital twins in the field of equipment monitoring.

With the development of industrial Internet of things and big data technology, using more abundant sensor data for model training and verification can significantly improve the performance of digital twins. This will not only enhance the prediction ability of gear fault and performance, but also promote the wide application and development of digital twin technology in the field of equipment monitoring. At the same time, considering that this study has not been combined with specific industrial deployment scenarios for downtime comparison or economic benefit evaluation, future research will be further extended to real manufacturing scenarios, and the operation and maintenance cost model and equipment downtime evaluation index will be introduced to further verify the engineering value and economic rationality of the system, so as to support the application value of the system in actual industrial scenarios. The above can be summarized as future research directions.

## Figures and Tables

**Table 1:** The number of meshes for each component.

Component	Number of Meshes
Lubricating Oil	185,900
Driving Gear	67,100
Driven Gear	97,500

**Table 2:** Parameters of Key Components.

Hardware	Specifications	Parameters
Three-Phase AC Frequency Converter Motor	Voltage: 220 V, Frequency: 50/60 Hz	Power: 0.55 kW, Maximum Speed: 1450 rpm
Magnetic Powder Clutch and Brake	—	Maximum Torque: 5 N·m
Gearbox	Oil-Immersed, Two N205 Rolling Bearings, Two S45C Gears	Large Gear: Module 2, Number of Teeth 75 Small Gear: Module 2, Number of Teeth 55
Full-Steel Anti-Vibration Base	Adjustable Pivot Position with ±2° Angular Adjustment Range	—

**Table 3:** R2 value of stress prediction model for driving wheel and driven wheel.

	Normal Gear	Broken Tooth Gear	Worn Gear	Pitting Gear	Average Value
Driving wheel stress R2	0.9341	0.9217	0.9434	0.9273	0.9355
Driven wheel stress R2	0.9469	0.9521	0.9634	0.9546	0.9479

**Table 4:** DRSN Network Structure Parameters.

Structural Layers	Layer Parameters			
	Convolution Kernels	Number	Stride	Output
Conv1	3 × 3 × 3	64	1	(64,H,W)
Conv2	64 × 3 × 3	64	1	(64,H,W)
Conv3	64 × 3 × 3	128	2	(128,H/2,W/2)
Conv4	128 × 3 × 3	256	2	(256,H/4,W/4)
Conv5	256 × 3 × 3	512	2	(512,H/8,W/8)
GAP	—	—	—	(512,1,1)
Flatten	—	—	—	512
FC	—	—	—	512

**Table 5:** Detailed description of gear types.

Type	Description
Health	Normal gear
Miss	Gear broken teeth missing teeth
Wear	Gear wear
Broke	Gear pitting

**Table 6:** Training results of different models.

Different Models	Recognition Accuracy/%	Accuracy Rate/%	Recall Rate/%	F1 Value/%	Loss Rate
ResNet	92.06	93.27	92.06	92.18	1 × 10^−4^
DenseNet	96.22	96.60	96.27	96.21	1 × 10^−4^
ConvNeXt	97.53	97.40	97.22	97.23	1 × 10^−5^
Proposed model	99.45	99.49	99.48	99.48	1 × 10^−5^

**Table 7:** R^2^ values of the stress prediction models for the driving wheel and the driven wheel.

	Normal Gear	Tooth Breakage Gear	Worn Gear	Pitting Gear	Average Value
R^2^ of the driving wheel stress	0.9452	0.9167	0.9413	0.9323	0.9339
R^2^ of the driven wheel stress	0.9557	0.9645	0.9373	0.9412	0.9497

## Data Availability

Data are contained within the article.
